# Novel flavin-containing monooxygenase protein FMO1 interacts with CAT2 to negatively regulate drought tolerance through ROS homeostasis and ABA signaling pathway in tomato

**DOI:** 10.1093/hr/uhad037

**Published:** 2023-02-28

**Authors:** Lulu Wang, Yinlian Zhou, Yin Ding, Chunrui Chen, Xueting Chen, Nini Su, Xingguo Zhang, Yu Pan, Jinhua Li

**Affiliations:** State Cultivation Base of Crop Stress Biology for Southern Mountainous land of Southwest University, Academy of Agricultural Sciences, Southwest University, Beibei, Chongqing 400715, China; Key Laboratory of Agricultural Biosafety and Green Production of Upper Yangtze River (Ministry of Education), College of Horticulture and Landscape Architecture, Southwest University, Chongqing 400715, China; State Cultivation Base of Crop Stress Biology for Southern Mountainous land of Southwest University, Academy of Agricultural Sciences, Southwest University, Beibei, Chongqing 400715, China; Key Laboratory of Agricultural Biosafety and Green Production of Upper Yangtze River (Ministry of Education), College of Horticulture and Landscape Architecture, Southwest University, Chongqing 400715, China; State Cultivation Base of Crop Stress Biology for Southern Mountainous land of Southwest University, Academy of Agricultural Sciences, Southwest University, Beibei, Chongqing 400715, China; Key Laboratory of Agricultural Biosafety and Green Production of Upper Yangtze River (Ministry of Education), College of Horticulture and Landscape Architecture, Southwest University, Chongqing 400715, China; State Cultivation Base of Crop Stress Biology for Southern Mountainous land of Southwest University, Academy of Agricultural Sciences, Southwest University, Beibei, Chongqing 400715, China; Key Laboratory of Agricultural Biosafety and Green Production of Upper Yangtze River (Ministry of Education), College of Horticulture and Landscape Architecture, Southwest University, Chongqing 400715, China; State Cultivation Base of Crop Stress Biology for Southern Mountainous land of Southwest University, Academy of Agricultural Sciences, Southwest University, Beibei, Chongqing 400715, China; Key Laboratory of Agricultural Biosafety and Green Production of Upper Yangtze River (Ministry of Education), College of Horticulture and Landscape Architecture, Southwest University, Chongqing 400715, China; State Cultivation Base of Crop Stress Biology for Southern Mountainous land of Southwest University, Academy of Agricultural Sciences, Southwest University, Beibei, Chongqing 400715, China; Key Laboratory of Agricultural Biosafety and Green Production of Upper Yangtze River (Ministry of Education), College of Horticulture and Landscape Architecture, Southwest University, Chongqing 400715, China; State Cultivation Base of Crop Stress Biology for Southern Mountainous land of Southwest University, Academy of Agricultural Sciences, Southwest University, Beibei, Chongqing 400715, China; Key Laboratory of Agricultural Biosafety and Green Production of Upper Yangtze River (Ministry of Education), College of Horticulture and Landscape Architecture, Southwest University, Chongqing 400715, China; State Cultivation Base of Crop Stress Biology for Southern Mountainous land of Southwest University, Academy of Agricultural Sciences, Southwest University, Beibei, Chongqing 400715, China; Key Laboratory of Agricultural Biosafety and Green Production of Upper Yangtze River (Ministry of Education), College of Horticulture and Landscape Architecture, Southwest University, Chongqing 400715, China; State Cultivation Base of Crop Stress Biology for Southern Mountainous land of Southwest University, Academy of Agricultural Sciences, Southwest University, Beibei, Chongqing 400715, China; Key Laboratory of Agricultural Biosafety and Green Production of Upper Yangtze River (Ministry of Education), College of Horticulture and Landscape Architecture, Southwest University, Chongqing 400715, China

## Abstract

Drought stress is the major abiotic factor that can seriously affect plant growth and crop production. The functions of flavin-containing monooxygenases (FMOs) are known in animals. They add molecular oxygen to lipophilic compounds or produce reactive oxygen species (ROS). However, little information on FMOs in plants is available. Here, we characterized a tomato drought-responsive gene that showed homology to FMO, and it was designated as *FMO1*. *FMO1* was downregulated promptly by drought and ABA treatments. Transgenic functional analysis indicated that RNAi suppression of the expression of *FMO1* (*FMO1*-Ri) improved drought tolerance relative to wild-type (WT) plants, whereas overexpression of *FMO1* (*FMO1*-OE) reduced drought tolerance. The *FMO1*-Ri plants exhibited lower ABA accumulation, higher levels of antioxidant enzyme activities, and less ROS generation compared with the WT and *FMO1*-OE plants under drought stress. RNA-seq transcriptional analysis revealed the differential expression levels of many drought-responsive genes that were co-expressed with *FMO1*, including *PP2Cs*, *PYLs*, *WRKY*, and *LEA*. Using Y2H screening, we found that *FMO1* physically interacted with catalase 2 (CAT2), which is an antioxidant enzyme and confers drought resistance. Our findings suggest that tomato *FMO1* negatively regulates tomato drought tolerance in the ABA-dependent pathway and modulates ROS homeostasis by directly binding to SlCAT2.

## Introduction

The adverse effects of various abiotic stresses on crop yield have become a major challenge in changing climates. Drought is a major environmental stress that dramatically affects agriculture worldwide [1]. Therefore, the molecular mechanism of drought-responsive genes needs to be characterized and understood to increase plant drought resistance. Plants develop drought stress tolerance through the integration of hormones and environmental signals that regulate different drought-responsive genes.

Hormones that regulate gene expression are essential to plants under drought stress [2]. As one of the most effective endogenous hormones in plants, abscisic acid (ABA) is a key regulator of the physiological processes of plants’ response to abiotic stresses [3]. For instance, ABA induces the accumulation of late embryogenesis abundant (LEA) protein and participates in the response of plants to drought stress [4]. LEA proteins widely exist in the cytoplasm and nucleus of plants and respond to various abiotic stress signals. ABA responses to drought stress occur through ABA-dependent and ABA-independent pathways [5]. ABA activates the SnRK2_S_ III response to drought in the ABA-dependent pathway [6]. In the ABA-dependent pathway, SnRK2s III, ABA receptor (ABA-PYR/PYL/RCAR) complexes, and protein phosphatases 2C (PP2Cs) are core components in ABA sensing [6]. SnRK2 III is the key positive regulator of the downstream signal of the ABA receptor. When drought stress occurs, ABA is largely stored in plant cells. Then, the ABA receptor competes with PP2Cs to combine with SnRK2s III; as a result, the inhibitory effect of PP2Cs on SnRK2s III is released [7–9]. Activated SnRK2s III can phosphorylate transcription factors, such as AREB or ABF, to increase the expression of abiotic stress response genes [6].

*NCED* (9-*cis*-epoxycarotenoid dioxygenase) is a crucial gene that regulates ABA synthesis [10]. Overexpression of *SlNCED1* can increase the concentration of ABA in leaves [11]. Aluminum can upregulate the expression of *ClNCED3* to accelerate the accumulation of ABA in roots and stomatal closure under aluminum stress [12]. The silencing of *NCED4* changes the ABA biosynthesis and signaling pathways related to gene expression [13].

Flavin-containing monooxygenases (*FMOs*) are a family of flavoenzymes that often contain an FAD and NADPH interaction motif [14]. They were discovered in mammals, and they function in catalyzation for oxygenation [15]*.* More *FMO* genes have been found in plants than in animals. For instance, 29 putative *FMO* genes have been discovered in *Arabidopsis*, whereas only five have been found in humans; meanwhile, only a single *FMO* gene has been identified in yeast [15]. Many recent reports have verified that *FMO* is involved in auxin biosynthesis, glucosinolate metabolism, and abiotic and biotic stresses. For instance, YUCCA proteins belong to a family of *FMOs* that catalyze critical steps in auxin biosynthesis, and they contribute to plant development and abiotic stress resistance [16–18]. The induction of *FMO_GS-OX2_* changes the glucosinolate profile of *Arabidopsis* leaves [19]. Overexpression of *FMO*_GS-OX1_ can cause a 5-fold increase in the levels of glucosinolates, which are broken down into isothiocyanates to enhance the cancer-preventive activity of cruciferous vegetables [20]. Furthermore, the induced expression of *FMO_GS-OX_* by glucose can increase endogenous ABA accumulation in *Arabidopsis thaliana*, and the trend is significantly inhibited in ABA-deficient (*aba2–1*, *aba3–1*) and ABA-insensitive (*abi1–1*, *abi2–1*) mutants [19]. *FMO* can use cysteine as a nitrogen source to produce nitrogenous aroma volatiles in tomato [21].

Tomato is one of the most important vegetable crops and plant models in the world. It has the characteristics of simple diploid inheritance, easy transformation, high reproductive efficiency, and short life cycle [22]. Tomato is often exposed to drought stress, which poses a critical threat to tomato growth and sustainable agriculture worldwide [23]. Transgenic methods have been successfully used to improve the drought resistance of tomato. Overexpression of the tomato WHIRLY transcription factor *SlWHY2* increases the drought and *Pseudomonas solanacearum* resistance of tobacco [24], and the transcription factor *SlbHLH22* enhances tomato salt and drought resistance [25]. Knockout of a protein kinase gene *SlOST1* reduces tomato drought resistance and exhibits a late flowering phenotype [26]. Moreover, tomato genes *SpPKE1*, *SlDREB*, *SlWRKY81*, *SlERF1*, and *SlWRKY70* play a positive role in drought resistance [27–30], and *SlbZIP38* and *HyPRP1* negatively regulate drought tolerance [31, 32]. In addition, knockout of *SlNPR1* reduces tomato drought resistance [33]. Therefore, discovering new drought resistance genes is important.

In this study, we studied tomato *FMOs*, such as *FMO1*, which is a drought-responsive gene screened via drought transcriptome analysis [34]. We demonstrated that inhibiting the expression of *FMO1* could significantly improve the drought tolerance of tomato, whereas overexpression of *FMO1* could make it increasingly sensitive. RNA-seq transcriptome analysis revealed that *FMO1* regulated the expression levels of ABA signaling and drought-responsive genes. *FMO1* physically interacted with catalase 2 (CAT2) via yeast-two-hybrid (Y2H) analysis, which can promote reactive oxygen species (ROS) scavenging, thereby conferring drought tolerance by maintaining ROS homeostasis. This work presents evidence on the critical role of *FMO1*, which negatively regulates drought tolerance by interacting with SlCAT2 and modulates the expression of drought-responsive genes in the ABA-dependent pathway.

## Results

### Gene features of FMO1

**Figure 1:**
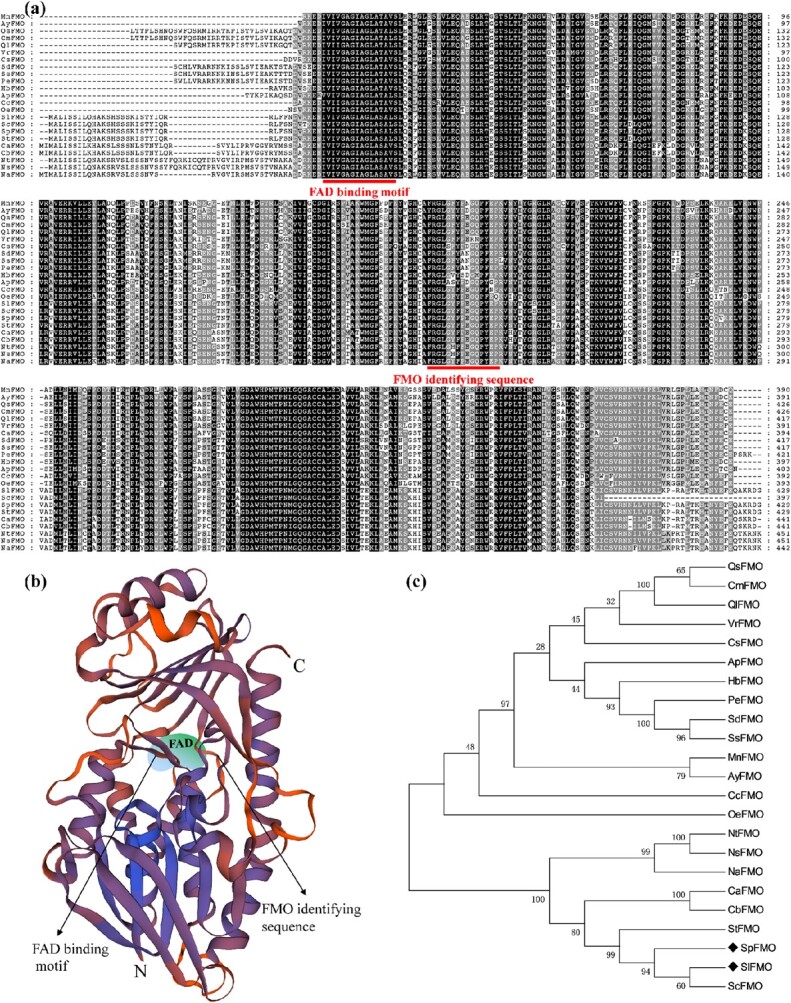
The gene features analysis of FMO1. **a** Sequence alignment of FMO1 protein amino acid. CLUSTAL alignment of deduced amino acid sequence of FMO1 and those of *Solanum tuberosum* (StFMO, XP_006359228.1), *Solanum chilense* (ScFMO, TMW81658.1), *Nicotiana attenuata* (NaFMO, XP_019252430.1), *Nicotiana tabacum* (NtFMO, XP_016505392.1), *Nicotiana sylvestris* (NsFMO, XP_009766505.1), *Capsicum annuum* (CaFMO, XP_016570429.1), *Capsicum baccatum* (CbFMO, PHT58423.1), *Coffea canephora* (CcFMO, CDP08357.1), *Populus euphratica* (PeFMO, XP_011047582.1), *Salix dunnii* (SdFMO, KAF9689763.1), *Quercus suber* (QsFMO, XP_023907324.1), *Vitis riparia* (VrFMO, XP_034691995.1), *Morus notabilis* (MnFMO, XP_010104831.1), *Salix suchowensis* (SsFMO, KAG5228806.1), *Quercus lobata* (QlFMO, XP_030945557.1), *Acer yangbiense* (AyFMO, TXG51210.1), *Castanea mollissima* (CmFMO, KAF3962019.1), *Hevea brasiliensis* (HbFMO, XP_021639125.1), *Olea europaea subsp. europaea* (OeFMO, CAA2980785.1), *Camellia sinensis* (CsFMO, XP_028083592.1), *Abrus precatorius* (ApFMO, XP_027360274.1). FAD binding motif ‘GxGxxG’ and FMO identifying sequence ‘FxGxxxHxxxY/F’ are underlined, respectively. **b** 3D protein structure representation of FMO1 protein. The ‘N’ and ‘C’ indicates the N and C terminus of amino acids, respectively. The 3D protein structure models are generated by the SWISS-MODEL(https://swissmodel.expasy.org/) homology. Two putative structural domains of FAD binding motif and FMO identifying sequence are labelled according to the crystal structures of FMO from *Schizosaccharomyces pombe* [14]. **c** Phylogenetic analysis of FMO1. The phylogenetic tree was constructed by MEGA 7.0 with neighbor-joining method at 1000 bootstrap replicate. The scale indicates branch length, and bootstrap values represent the divergence of each branch.

The full-length cDNA of the tomato *FMO1* sequence was 1293 bp and encoded with 430 amino acids (Fig. 1a). The most homologous proteins were retrieved through NCBI pBLAST FMO1. All of the highly homologous *FMOs* were from plants. All *FMO1* sequences showed high conservation (Fig. 1a), suggesting that *FMO1* may have similar functionality in different species. Compared with previously reported structures of *FMOs* [14, 15], the 3D protein structure of *FMO1* was generated by SWISS-MODEL homology. Tomato *FMO1* was composed of two conservated binding domains. Located close to the N-terminus of the protein was the FAD-binding ‘GxGxxG’ motif, and the *FMO*-identifying motif ‘FxGxxxHxxxY/F’ was located near the center of the protein (Fig. 1a and b). However, the less well-conserved NADP-binding motif was not found in all the plant *FMOs*. A phylogenetic tree was constructed based on the protein sequences of *FMO1* and the retrieved highly homologous plant *FMOs*. It showed that all Solanaceae *FMOs* clustered together (Fig. 1c).

### Expression patterns of *FMO1* under plant hormone and abiotic stress treatment

**Figure 2:**
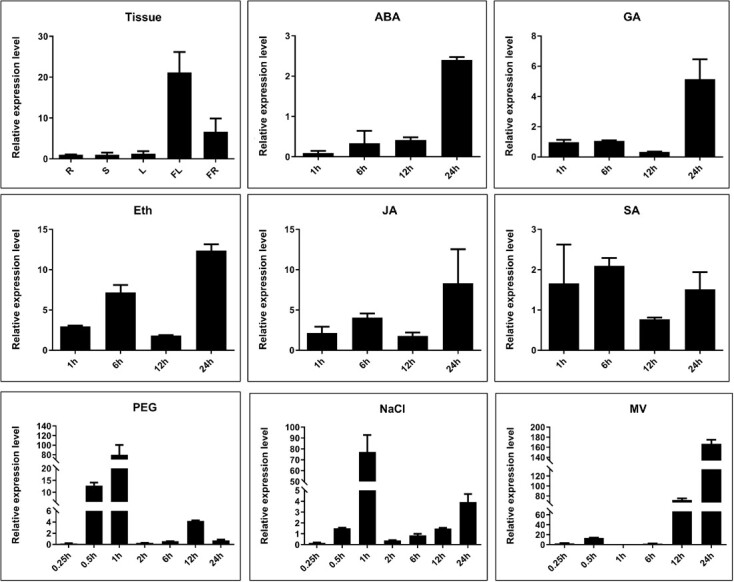
Expression pattern analysis of *FMO1.* Tissue Expression Pattern (R:Root；S:Stem；L:Leaf；FL:Flower；FR:Fruit), in response to the phytohormones (ABA, GA, Eth, JA, and SA) treatment and under various abiotic treatments (PEG, NaCl, MV) (time: h). All samples were collected at the indicated time points. Expression of treated WT plants was compared with that in untreated plants after normalization of values with reference to the tomato *β-actin* gene and is presented as the relative expression level. All samples were collected at the indicated time points from three biological replicates in each treatment. Error bars indicate the standard error (SE) of three replicates.

The tissue-specific expression of *FMO1* was analysed by quantitative reverse transcription polymerase chain reaction (qRT-PCR). *FMO1* was expressed in all the detected tissues, among which the expression in the flowers was the highest, followed by that in the fruits and leaves (Fig. 2).

ABA, gibberellic acid (GA), ethylene (Eth), jasmonic acid (JA), and salicylic acid (SA) are important response hormones and signal transduction factors when plants encounter abiotic stress [35]. To determine whether these hormones regulate the expression of *FMO1*, we investigated the effect of exogenous plant hormone application on the expression of *FMO1* via qRT-PCR. We found that the expression of *FMO1* was rapidly downregulated at 1 h and at 6 h or 12 h. Then, it was upregulated at 24 h under ABA and GA treatments. However, under the Eth and JA treatments, the *FMO1* expression was induced 1 h after treatment and was the highest at 24 h. After SA treatment, the *FMO1* expression was also upregulated at 6 h (Fig. 2). These results indicate that *FMO1* was downregulated by ABA and GA but upregulated by Eth, JA, and SA.

The expression of *FMO1* in the various stress treatments was determined to confirm if *FMO1* participates in abiotic stress. *FMO1* showed identical expression profiles under the drought/PEG and salt/NaCl treatments. It was rapidly downregulated at 0.25 h and showed elevated transcripts for up to 1 h. Then, it reverted to a low expression level. Moreover, under the MV (methyl viologen; induced oxidative stress) treatment, the expression of *FMO1* was rapidly downregulated at 0.25 h up to 6 h after treatment; elevated transcripts were obtained 12 h later (Fig. 2). These results illustrate that *FMO1* responded to drought stress and was likely induced by salt and oxidation. Moreover, it appeared to be a negative regulator at the early stage of the abiotic stress response.

### Inhibiting the expression of *FMO1* regulates drought resistance, and overexpression made it increasingly sensitive

**Figure 3:**
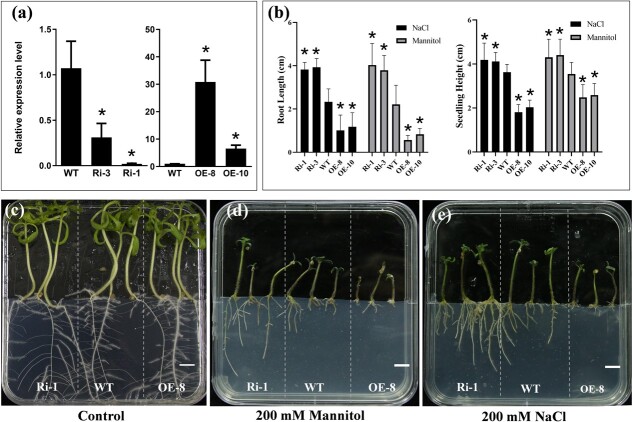
Determination of drought and salt tolerance of *FMO1* transgenic plants. **a** Quantitative real-time PCR analysis of expression level of *FMO1* transgenic tomato (WT: wild type; OE-8, OE-10: *FMO1* overexpressing lines；Ri-1, Ri-3:*FMO1* RNAi silencing lines). **b** Root length and seedling height of *FMO1* transgenic tomato and WT plants in mannitol and salt stress treatment. **c** Phenotypic differences between *FMO1* transgenic lines and WT tomatoes on MS medium after sowing 12 days. **d** Phenotypic differences between *FMO1* transgenic lines and WT tomatoes on MS medium under simulated drought treatment after sowing 12 days. **e** Phenotypic differences between *FMO1* transgenic lines and WT tomatoes on MS medium under simulated NaCl treatment after sowing 12 days. The simulated drought and salt stress assays were performed two times independently with similar results and one representative result is shown, and other transgenic lines assays were shown in Fig. S1 (see online supplementary material); the significant difference between the transgenic lines and the WT (*P*<0.05, ANOVA, Tukey test) is marked as *. The error bars indicate the SEs (*n* = 15). Scale Bars: 1 cm.

Ten *FMO1*-OE and 8 *FMO1*-RNAi lines were generated to investigate the function of *FMO1*. The qRT-PCR screening results showed two *FMO1*-OE lines (OE-8 and OE-10) and two *FMO1*-RNAi lines (Ri-1 and Ri-3) with significant differential expression compared with the wild-type (WT) lines (Fig. 3a). These transgenic lines were selected and grown on MS media by drought and salt stress treatments for functional analysis. Mannitol, a compatible solute accumulated in plants, is a substance that produces osmotic stress; it can be used as a substitute to simulate osmotic stress in drought stress experiments [36]. Under the control MS media, the growth phenotypes of WT, RNAi, and OE plants showed no significant differences (Fig. 3c; Supplemental Fig. S1, see online supplementary material). However, under mannitol stress, the growth and root length of the *FMO1*-OE lines were smaller than those of the WT lines, but the growth of the *FMO1*-RNAi lines was much higher than that of WT. The root lengths of the *FMO1*-RNAi lines were larger than those of WT (Fig. 3b and d). Similarly, when treated with 200 mM NaCl, the root length of the *FMO1*-OE lines was smaller than that of the WT lines, but the root length of the *FMO1*-RNAi lines was larger than that of WT (Fig. 3b and e). These results reveal that inhibiting the expression of *FMO1* could improve the drought and salt resistance of tomato.

**Figure 4:**
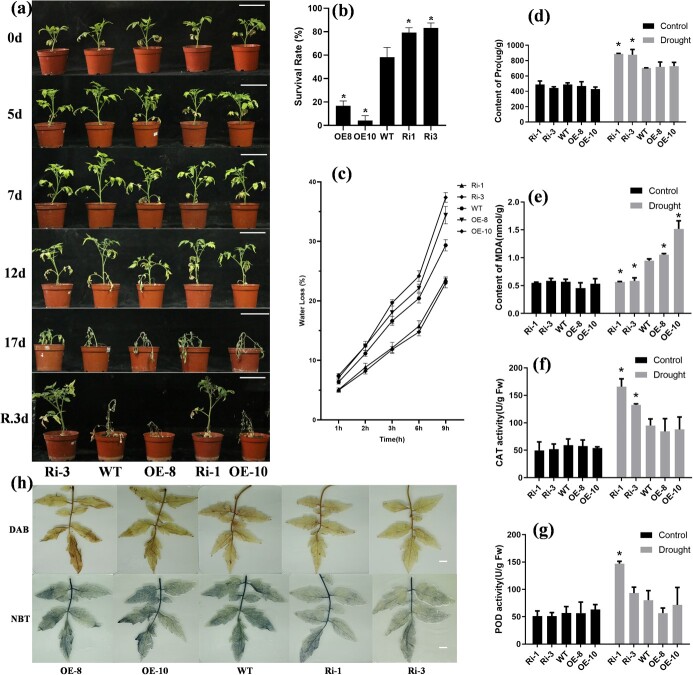
*FMO1* negatively regulates drought tolerance in tomato. **a** Phenotypes of *FMO1* transgenic and wild-type tomato under drought treatment (WT: wild type; OE-8, OE-10: *FMO1*overexpressing lines; Ri-1, Ri-3: *FMO1* RNAi silencing lines). **b** Survival rate of WT and *FMO1* transgenic plants scored after recovery. **c** Water loss rate of the OE, Ri and WT plants represented as a percentage of the initial fresh weight (*n* = 6). **d** Changes in proline content of WT and *FMO1* transgenic plants under normal growth and drought stress. **e** Changes in MDA content of WT and *FMO1* transgenic plants under normal growth and drought stress. **f**–**g** Changes in CAT and POD activities of WT and *FMO1* transgenic plants under normal growth and drought stress. **h** Tissue staining of WT and *FMO1* transgenic plants under drought stress using DAB and NBT. The drought tolerance testing was performed three times independently with similar results and one representative result is shown. The error bars indicate the SEs of means of three biological replicates (eight plants for each replicate). Statistical tests were performed by two-way ANOVA, followed by Tukey’s test (*P* < 0.05). Scale bars: (**a**) 10 cm; (**g**) 1 cm.

To further verify the effect of *FMO1* expression on the regulation of drought tolerance, we grew the WT and *FMO1* transgenic lines in soil under normal watering conditions and applied drought treatment up to the five-leaf stage. As shown in Fig. 4a, before the drought treatment, the WT and transgenic lines grew well. After the 7-day drought treatment, the leaves of the WT and *FMO1*-OE lines began to wilt, and no clear change was observed in the *FMO1*-RNAi lines. After 12-day drought treatment, the WT and *FMO1*-OE lines wilted more seriously than the *FMO1*-RNAi lines did. After 17-day drought treatment followed by a 3-day recovery process, the WT and *FMO1*-OE lines were still wilted and could not survive, but most of the *FMO1*-RNAi lines resumed their growth. After 17-day drought treatment, the survival rate of the *FMO1*-RNAi lines was >80%, which was considerably higher than the survival rate of the WT lines. Meanwhile, the survival rate of *FMO1*-OE was less than 20% (Fig. 4b). Moreover, the water loss detected in the *FMO1*-RNAi lines was smaller than that in the WT and *FMO1*-OE lines (Fig. 4c). This result indicates that inhibiting the expression of *FMO1* increased drought tolerance, whereas overexpression of *FMO1* decreased drought tolerance and increased sensitivity.

### Inhibited expression of *FMO1* improved the drought resistance of tomato by accumulating abundant proline and regulating ROS homeostasis

The capability of plants to cope with drought stress is closely related to the accumulation of proline (Pro) in plants, and the concentration of Pro is usually higher in stress-tolerant plants than in stress-sensitive plants. Moreover, excessive ROS production under drought has been reported to increase the malondialdehyde (MDA) content, an indicator of oxidative damage. Thus, in this study, the Pro and MDA contents of WT and transgenic plants under normal watering and drought stress conditions were investigated. The Pro and MDA contents of the WT and transgenic plants showed no significant difference under normal watering. After drought treatment, the Pro contents of the *FMO1*-RNAi lines were higher than those of the WT and *FMO1*-OE lines. On the contrary, the MDA contents of the *FMO1*-RNAi lines were lower than those of the WT and *FMO1*-OE lines (Fig. 4d and e).

ROS induced by abiotic stress directly causes plant cell damage. To reduce the effects of ROS, plants develop low-molecular-weight antioxidants and reactive oxygen scavenging enzymes, including peroxidase (POD) and catalase (CAT). Therefore, the activities of CAT and POD in the WT and transgenic plants were monitored before and after drought stress in the current study. The CAT and POD activities of the *FMO1*-RNAi lines were higher than those of the WT and *FMO1*-OE lines (Fig. 4f and g). This finding is consistent with the observation of mature leaves via histochemical staining by 3,3′-diaminobenzidine (DAB) and nitroblue tetrazolium chloride (NBT) after drought stress. The distribution of H_2_O_2_ and O^2−^*in situ* in the leaves of the *FMO1*-RNAi lines was less intense than that in the WT and *FMO1*-OE lines, especially at the top of the tomato leaves (Fig. 4h). This finding indicates that inhibiting the expression of *FMO1* enhanced tomato drought tolerance via increased accumulation of Pro, improvement of the activities of CAT and POD, and MDA content reduction.

### *FMO1* silencing improved tomato drought tolerance by regulating the ABA-dependent pathway and drought stress-related genes

To further study the molecular mechanism of *FMO1* gene regulation during drought stress, RNA sequencing was performed to analyse the genes and pathways regulated by *FMO1*. A total of 12 cDNA libraries, including WT and *FMO1* transgenic lines (OE and Ri), were present in DT and CK (DT: drought treatment and CK: control). The cDNA library of each sample produced about 39.67–46.11 million reads, and the correct event rate of sequencing reached 98% (Table S1, see online supplementary material). The sequences were aligned to the tomato-released genome ITAG3.0, as shown in Table S2 (see online supplementary material). Approximately 38.57–44.95 million reads were mapped to the tomato genome, accounting for 97.1% of the total reads on the average. More than 95% of the clean reads were uniquely located in the tomato genome, indicating that the RNA-seq results were reliable.

**Figure 5:**
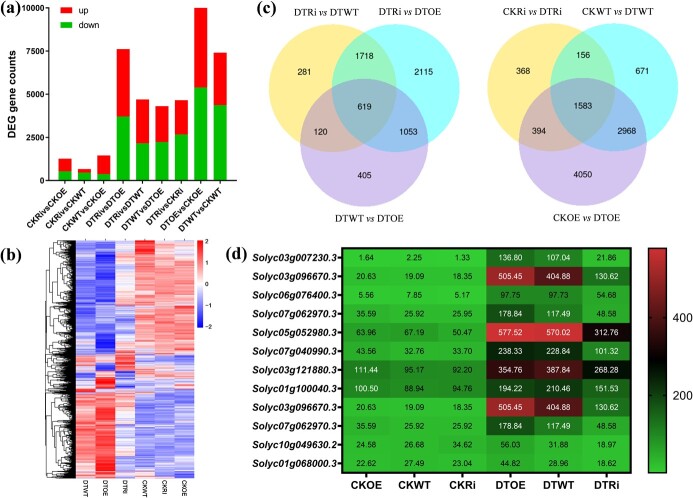
Differential expressed genes in *FMO1* transgenic plants upon drought stress as revealed by RNA-seq. **a** The number of differentially expressed genes in *FMO1* transgenic plants (OE: Transgenic lines overexpressing *FMO1*; Ri: *FMO1* RNAi silencing lines) compared with their respective wild type (WT) under DT and CK conditions (DT: Drought and CK: Control). **b** Heat cluster patterns of differentially expressed mRNA transcripts in all experimental Differential Gene Venn Diagram. **c** Venn diagram showing the number of differentially expressed genes in *FMO1* OE, WT and Ri with DT or DT vs CK. **d** Heat map of *PP2Cs* in GO analysis, showing their transcriptional abundance in WT and *FMO1* transgenic lines (OE and Ri) present at DT and CK. The colored bar indicates FPKM values. The RNA-seq results of OE and Ri under DT and CK conditions were based on two biological replicates per treatment.

Next, |log_2_ (FoldChange)| > 1 and padj ≤0.05 were used as criteria to screen genes with significantly different expression levels and to perform statistical analysis. As shown in Fig. 5a, under CK watering conditions, fewer differentially expressed genes (DEGs) were present among WT, Ri, and OE compared with the DT conditions. The heat clusters of numerous DEG expression changes are presented in Fig. 5b. For WT, Ri, and OE under CK and DT, the colors of the heatmap expression levels differed significantly between DT and CK, indicating that the expression of numerous genes was regulated by *FMO1*, especially under drought conditions. Many genes responded to drought, and the expression of *FMO1* could regulate many genes under drought stress.

**Figure 6:**
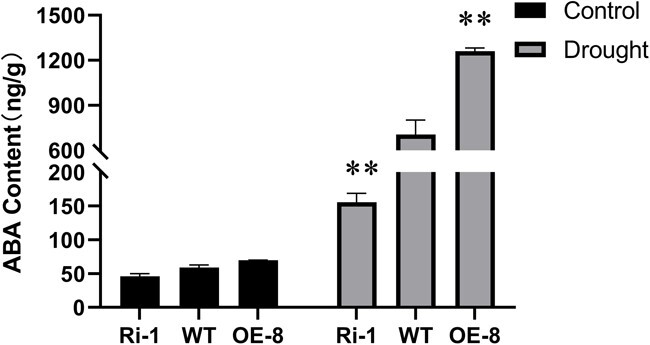
ABA content of *FMO1* OE, Ri lines and WT present at control and drought. The significantly difference between the transgenic lines and the WT (*P*<0.001, ANOVA, Tukey test) is marked as ^**^.

The Venn diagram analysis revealed 619 and 1583 common DEGs in Ri, WT, and OE under DT intercomparison and DT versus CK, respectively (Fig. 5c). We classified these differential genes to determine the key genes and metabolic pathways that *FMO1* regulated during drought. The expression levels of several *PP2Cs* increased considerably after drought treatment, and they were higher in the *FMO1*-OE plants than in the *FMO1*-RNAi and WT plants (Fig. 5d), indicating that *FMO1* and *PP2C*s were co-expressed. This result also implies that *FMO1* participated in the ABA-dependent pathway by modulating *PP2C* transcription to improve plant drought resistance. Gene ontology (GO) analysis further revealed that the DEGs mainly assembled during ion transport, photosynthesis, and plant hormone signal transduction (Fig. S2, see online supplementary material). This finding is consistent with the KEGG pathway enrichment results (Fig. S3, see online supplementary material). The shared DEGs under drought stress were enriched in the MAPK signaling pathway, which mainly included the ABA receptor *PYR/PYL* (Fig. S4a, see online supplementary material), *PP2C*s (Fig. 5d), photosynthesis, plant hormone signal transduction, and carbon metabolism (Fig. S3, see online supplementary material). In the plant hormone synthesis and signal transduction pathway, except for ABA sensing and the signaling core component *PP2C*s, the DEGs were enriched in the ABA synthesis pathway, e.g. the ABA biosynthesis gene *NCED1* (Table S3, see online supplementary material).

To confirm whether *FMO1* is involved in the ABA pathway, the ABA contents of *FMO1*-OE, *FMO1*-RNAi, and WT under DT or CK were determined. As shown in Fig. 6, the ABA contents of the WT and transgenic plants showed no significant difference under normal watering. However, after drought treatment, the ABA contents of the *FMO1*-OE lines were significantly higher than those of the WT and *FMO1*-Ri lines. The ABA content of WT was also significantly higher than that of the *FMO1*-Ri lines. According to these observations, the *FMO1* gene possibly participated in ABA-dependent signaling and negatively regulated the drought resistance of tomato.

The enriched genes in the GO term regulation of transcription showed stress-related DEGs, including cytoplasmic ascorbate peroxidase 1, monodehydroascorbate reductase 1, peroxidase, catalase isozyme 2 isoform X1, superoxide dismutase, and stress-related proteins, and other oxidative stress-related genes, transporters, and transcription factors (Table S4, see online supplementary material). The WRKY transcription factors were co-expressed with *FMO1* (Fig. S4b, see online supplementary material), and most of the co-expressed genes were negatively regulated by *FMO1*. However, the LEA hydrophilic protein genes, which have been implicated in the stress responses of many plants, downregulated the *FMO1*-RNAi line after drought stress treatment and were positively regulated by *FMO1* (Fig. S4c, see online supplementary material)*.* To further verify the reliability of the transcriptome results, we selected 12 DEGs, such as *PP2C*, that were related to the ABA biosynthesis gene *NCED1* and abiotic stress genes for real-time PCR verification (Table S3, see online supplementary material). The quantitative results (Fig. S5, see online supplementary material) of this experiment were in good agreement with the RNA-seq data (Table S3, see online supplementary material). Our results strongly support the idea that tomato *FMO1* negatively regulates drought tolerance by regulating the ABA synthesis and signal transduction pathway and drought stress-related genes.

**Figure 7:**
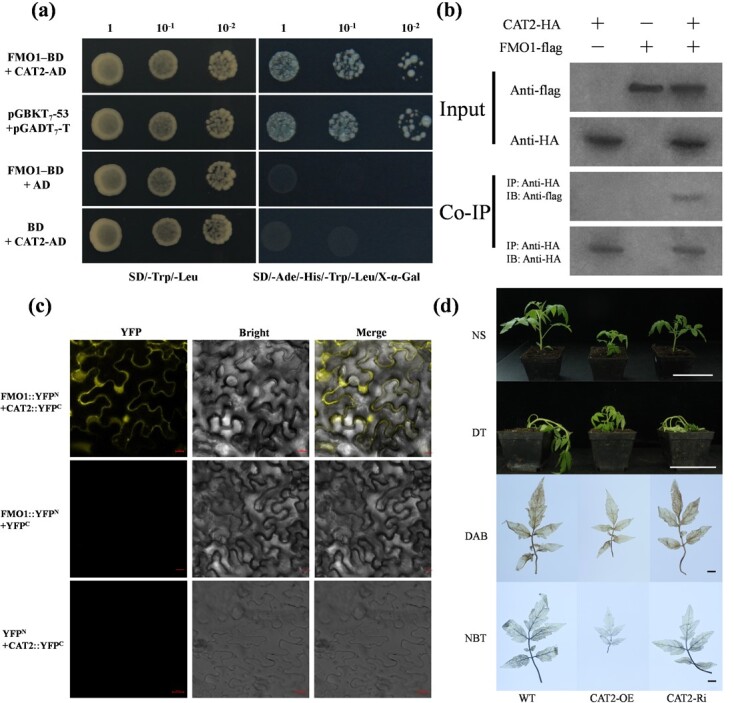
SlFMO1 interacts with SlCAT2 *in vitro* and *in vivo* and functional characterization of *SlCAT2*. **a** Yeast two-hybrid assays for the interactions between FMO1 and CAT2. Yeast growth on SD/−Leu/−Trp and SD/−Ade/-His/−Leu/−Trp/X-α-Gal solid medium is shown. N: Negative control， pGBKT_7_-Lam + pGADT_7_-T; P:Positive control， pGBKT_7_–53 + pGADT_7_-T. **b** Co-IP assays showing the interaction of FMO1 and CAT2 in *N. benthamiana* leaves. Anti-HA and anti-FLAG antibodies were used for immunoprecipitation, respectively. The full blot images of Co-IP was shown in supplemental Fig. S7. **c** BiFC assay of FMO1 interaction with CAT2 in *N. benthamiana* leaves. The C-terminal half of GFP was fused to CAT (CAT2:: YFP^C^), and the N-terminal half of GFP was fused to FMO1 (FMO1::YFP^N^). The expression of FMO1::YFP^N^ + YFP^C^ and YFP^N^ + CAT2::YFP^C^ were used as control. Bar = 50 μm. **d** Phenotype and drought resistance analysis of *SlCAT2* overexpression (OE) and RNAi (Ri) transgenic lines under drought (DT) and no stress (NS) condition. Tissue staining of WT and *SlCAT2* transgenic plants under drought stress using DAB and NBT. Scale bars: upper 10 cm; bottom 1 cm.

### FMO1 interacted with CAT2 to confer drought resistance

Y2H screening of interacting proteins was performed to further analyse how *FMO1* regulated drought tolerance. *FMO1* was ligated to pGBKT7 to produce a pGBKT_7_-*FMO1* (pBD-*FMO1*) fusion vector. Then, we used pBD-*FMO1* to screen the tomato cDNA yeast library matchmaker Gold Y2H system. The positive clones were stained with X-α-Gal. Approximately 50 positive clones were selected and sequenced, and some proteins that possibly interact with *FMO1* were obtained, including SlCAT2 (Solyc12g094620) and stress-related proteins (Table S5, see online supplementary material). The deduced amnio acid sequence of SlCAT2 shared 89%, 86%, and 86% similarity with AtCAT2 (AT4G35090), AtCAT1 (AT1G20630), and AtCAT3 (AT1G20620), respectively (Fig. S6, see online supplementary material). To verify the reliability of the results, we cloned the putative interacting protein SlCAT2 gene for point-to-point verification. When the yeast contained tomato *FMO1* and CAT2 protein, it grew and turned blue on the medium of SD/−Ade/His/−Leu/−Trp (containing X-α-Gal) (Fig. 7a), indicating that *FMO1* interacted with SlCAT2 in the yeast. Furthermore, we used co-immunoprecipitation (Co-IP) and bimolecular fluorescence complementation (BiFC) to verify the results. The Co-IP assays demonstrated the existence of *FMO1*/CAT2 protein complexes in *Nicotiana benthamiana* leaves (Fig. 7b), and the BiFC results showed that when *FMO1*: YFP^N^ and CAT2: YFP^C^ were co-expressed in the *N. benthamiana* leaves, the leaves showed yellow fluorescence, which was not observed in the empty vector (Fig. 7c). This result further proves that *FMO1* and CAT2 interacted with each other.

To determine if CAT2 is an antioxidant enzyme and confers drought resistance, we performed transgenic function analysis by using *CAT2* OE and Ri plants. As presented in Fig. 7d, *CAT2*-OE showed a dwarfing phenotype under normal conditions. After drought stress, the *CAT2*-Ri and WT plants wilted, but the *CAT2*-OE lines grew well. The DAB and NBT histochemical staining results of mature leaves after drought stress revealed that the distribution of H_2_O_2_ and O^2−^*in situ* in the leaves of the *CAT2*-OE lines was less intense than that in the leaves of the WT and *CAT2*-Ri lines (Fig. 7d). This result indicates that *CAT2* positively regulated drought resistance by scavenging ROS.

## Discussion

### Tomato abiotic response gene *FMO1* is widespread and highly conserved in plants

We identified a novel *FMO* through tomato drought transcriptome data analysis. A CLUSTAL alignment of *FMOs* in plants, which was highly homologous with tomato *FMO1* sequences, was present, indicating that the *FMOs* were widespread in the plants. These findings imply that *FMO1* may have similar functions in different species. In *Schizosaccharomyces pombe*, *FMO* is composed of FAD binding its complex with NADPH and an enzyme–substrate complex [14]. The plant *FMOs* in the present study shared highly conserved *FMO*-identifying and FAD-binding motifs but no NADPH interaction domain (Fig. 1a and b). These findings differ from those obtained for the pepper *FMO* gene *Bs3*, which contains an NADPH binding site in the middle of amino acid residues [37].

According to the current tissue expression analysis of *FMO1* in tomato, the gene was highly expressed in the fruits, flowers, and leaves. *FMO1* was downregulated at the early stage of drought stress (Fig. 2). *FMO1* probably has a crucial function in the process of drought resistance during tomato growth, and whether it also improves the flower and fruit preservation rate in tomato after drought stress remains to be further studied. Moreover, we detected the expression of *FMO1* response to different plant hormones through qRT-PCR. The expression of *FMO1* was rapidly downregulated at 1 h under ABA treatment but constitutively upregulated by Eth and JA (Fig. 2). JA and ABA are phytohormones that activate the defense system to enhance plant resistance to abiotic stresses [35]. ABA and Eth production increases during drought stress in tomato plants in response to biotic and abiotic stresses [38]. Therefore, *FMO1* regulates ABA, Eth, and JA signaling and is probably involved in hormone crosstalk and drought stress.

### *FMO1* serves as a negative regulator of drought stress by modulating antioxidant-related enzymes and osmotic substances

Drought stress seriously restricts the growth of plants and therefore severely affects the yield and quality of crops. As a result, improvement of drought resistance has become a long-term goal in crop breeding. Recently, several genes have been successfully applied to increase the drought resistance of tomato. *SlbZIP1* modulates an ABA-mediated pathway that contributes to drought stress tolerance [39], and overexpression of *SlMAPK3* results in increased tolerance to Cd^2+^ and drought in tomato [40]. Overexpression of *SlNAC2* in tobacco improves plant survival and recovery, which in turn increases drought resistance. *SlWRKY81* attenuates stomatal closure and subsequent drought tolerance by reducing nitric oxide accumulation [28]. Increased *SlBRI1* expression levels reduce tomato drought resistance due to increased brassinosteroid signaling intensity [25], and overexpression of *SlERF.B1* results in tomato plants that are less tolerant to drought and mannitol stress [41]. Many *FMO*s have been investigated in yeast and mammals, and relevant research has shown that their main function is to add molecular oxygen to lipophilic compounds or to produce ROS [14, 15]. However, until recently, the function of *FMOs* in plants has remained elusive.

In this study, we performed transgenic functional analysis to verify whether *FMO1* participates in the abiotic resistance of tomato. By comparing the phenotypic differences among *FMO1*-OE, *FMO1*-Ri, and WT plants after drought stress, we found that the drought tolerance of the *FMO1*-Ri plants was higher than that of the *FMO*-OE and WT plants (Figs 3d and 4a). Thus, tomato *FMO1* negatively regulates drought tolerance, and it is unlike the first plant-identified *FMO* protein YUCCAs, which positively regulate muti-abiotic stress tolerance, including drought, aluminum stress, heat stress, and shade [18]. YUCCAs share similar properties and typical structures with mammalian *FMO*, which contains two conserved ‘GxGxxG’ sequences for FAD and NADPH binding. However, in this study, the tomato amino acid of *FMO1* had very low homology with YUCCAs and mammalian *FMOs*, and the homology search and sequence alignment results showed that *FMO1* was specific to plants. Moreover, tomato *FMO1* only possessed a FAD-binding motif near the N-terminus, and there was no NADPH binding motif in the middle region (Fig. 1a and b). Our observations led us to conclude that tomato *FMO1* is a novel *FMO* that is a negative regulator of drought tolerance.

Drought stress alters cellular redox homeostasis and generates ROS, including H_2_O_2_, hydroxyl radicals (OH^−^), and superoxide radical (O^2−^) [42]. Thus, ROS scavenging can protect plants against drought stress [43]. To minimize drought-induced oxidative damage, plants have various physiological functions, including antioxidant metabolism and osmotic adjustment [44]. Therefore, we measured the relevant physiological indicators of the *FMO1*-OE, *FMO1*-Ri, and WT plants before and after drought stress. The Pro content of the *FMO1*-RNAi lines was higher than that of the WT and *FMO1*-OE lines under drought stress (Fig. 4d), but the MDA content in the WT and *FMO1*-OE lines increased (Fig. 4e). *FMO1* silencing conferred tolerance to drought by adjusting the Pro content and reducing the level of membrane lipid peroxidation. Similarly, under drought stress treatment, we found that the activities of CAT and POD in the *FMO*-RNAi lines were higher than those in the WT plants (Fig. 4f and g), and the leaves of the *FMO*-RNAi lines exhibited lower ROS accumulation than those of the WT and *FMO1*-OE lines after visualization by DAB and NBT histochemical staining (Fig. 4h). Similar trends have been observed for *Arabidopsis*, where *AtFMO1* is related to ROS production, especially H_2_O_2_; it is involved in DNA damage and root meristem [45]. Over-accumulation of ROS caused by drought stress results in membrane lipid peroxidation, but some enzymatic antioxidants, such as CAT, play an essential role in eliminating ROS [46]. Overall, we posit that *FMO1* negatively regulates drought tolerance by accumulating abundant antioxidant-related enzymes and osmotic substances to reduce ROS damage.

### Molecular mechanism of *FMO1* regulating drought resistance of tomato

Although ROS is involved in many biological processes by acting as signaling molecules, plant cells can suffer from oxidative damage due to excessive ROS accumulation [47]. Catalases exist in almost all living organisms and are known as the most effective ROS scavenger because of their high catalytic decomposition capacity toward H_2_O_2_, which plays important roles in plant abiotic stresses [47]. In *Arabidopsis*, three CAT genes, namely, *AtCAT1*, *AtCAT2*, and *AtCAT3*, have been characterized [48]. *AtCAT1* is mediated by MAPK cascades, and its expression is upregulated by ABA, which suppresses ROS accumulation. *AtCAT2* activity is necessary for long-term heat tolerance, and CAT2 (*AtCAT2* mutant) accumulates abundant H_2_O_2_ by reducing leaf catalase activity. Under drought conditions, *AtCAT3* plays a vital role in H_2_O_2_ homeostasis and stomatal regulation mediated by ABA. In this study, tomato SlCAT2 was found to interact with FMO1 (Fig. 7), and the expression of *FMO1* affected CAT and POD activities to reduce the membranous peroxidation damage (Fig. 4f and g). SlCAT2 also interacted with SlMSR B2, which is involved in drought resistance by promoting ROS scavenging [49]. The OE of *SlCAT2* resulted in a dwarfing phenotype and enhanced drought resistance by scavenging ROS (Fig. 7d). Altogether, our results strongly support the notion that *FMO1* is involved in tomato drought resistance by directly modulating SlCAT2 and regulating the expression of other genes related to ROS-scavenging enzymes’ expression (Table S3, see online supplementary material) or by affecting enzymatic activities (Fig. 4f and g). Thus, exploration of the detailed mechanism of whether and how tomato *FMO1* catalyzes the oxygenation of CAT2 under drought stress is worthy of attention.

The RNA-seq results revealed that the number of DEGs in WT, *FMO1*-OE, and *FMO1*-Ri lines under drought conditions increased significantly compared with that under normal growth conditions (Fig. 5). The DEGs mainly assembled during ion transport, photosynthesis, plant hormone signal transduction, carbon metabolism, and MAPK signaling (Fig. 5d; Figs. S2 and S3, see online supplementary material), which are crucial processes in plants coping with drought stress. The MAPK signaling pathway is the core of the cell signal transduction system [50]. This pathway is crucial for plant responses to abiotic stresses and hormone signal transduction [50]. Through RNA-seq analysis, we found that the shared DEGs among WT, Ri, and OE in the MAPK signaling pathway included ABA receptor, *PP2Cs*, and some protein kinases (Fig. S3, see online supplementary material). In the plant hormone signal transduction pathway, aside from *PP2Cs*, the key gene in ABA biosynthesis, *NCED1*, was induced in the *FMO1*-OE plants and inhibited in the *FMO1*-Ri plants. At least 12 *PP2C*s were co-expressed with *FMO1* under drought stress (Fig. 5d). *PP2Cs* are responsible for negative regulation of ABA signaling via dephosphorylation of SnRK2 [51]. Thus, *PP2Cs* are insensitive to ABA [52], and overexpression of *PP2Cs* results in sensitivity to drought stress [53]. SnRK2 phosphorylates various substrates, including AREB/ABFs, FBH3/AKS1, and SNS1 in the ABA-dependent pathway, and modulates their activities [5]. Recent studies have suggested that TaWRKY1 in wheat plays a crucial role in drought tolerance and the ABA-dependent pathway [54]. In our study, the WRKY transcription factors were co-expressed with *FMO1* (Fig. S4b, see online supplementary material), and most of these co-expressed genes were negatively regulated by *FMO1.* These findings, together with our result that *FMO1* negatively regulates drought tolerance (Fig. 4), led us to conclude that *FMO1* modulates the ABA signal by regulating *PP2Cs* and other stress-resistant genes, such as WRKY transcription factors, to cope with drought stress in the ABA-dependent signaling pathway.

*NCED1* was downregulated in the *FMO1* RNAi lines and upregulated in the *FMO1* OE plants (Table S3, see online supplementary material). *NCED* encodes a key enzyme during ABA biosynthesis to control plant accumulation of ABA [55]. For instance, the *NCED3* mutant shows decreased ABA content, which reduces drought resistance. On the contrary, overexpression of *NCED3* increases the ABA content and drought resistance [56]. This finding is consistent with our observation that ABA was abundantly accumulated in the *FMO1* OE plants and significantly decreased in the *FMO1* Ri plants (Fig. 6). A low ABA concentration inhibits *PP2C* activity and promotes root growth and hydrotropism [57]. Therefore, we suggest that the low ABA content in the *FMO* RNAi plants (Fig. 6) resulted in enhanced root growth under mannitol and NaCl stresses (Fig. 3d and e) and conferred drought tolerance (Fig. 4) by downregulating the expression of *PP2Cs* (Fig. 5d). The low accumulation of ABA in the *FMO* RNAi plants might be due to the feedback of *PP2C* downregulation by activating the ABA signaling pathway. Thus, we speculate that *FMO1* negatively regulates drought resistance primarily by affecting ABA signaling then increasing ABA synthesis. Moreover, the pepper *FMO* gene *Bs3* triggers plant cell death, impairs yeast growth, and produces H_2_O_2_, thereby promoting ABA signaling via the inactivation of the negative regulator *PP2C*s [37, 58]. This finding helps explain why the *FMO1* RNAi-inhibited plants in our study accumulated less H_2_O_2_ and had a low expression of *PP2Cs*. Our results strongly support the idea that *FMO1* regulates ABA signaling and CAT-mediated antioxidant pathways to cope with drought resistance in tomato. Many clues show that the two pathways are not independent. A previous study has reported that the content of H_2_O_2_ decreases when ABA production is reduced, which is closely related to the activity of catalases [59]. H_2_O_2_ plays a vital intermediary function in the ABA signal pathway, thereby inducing the expression of *CAT1* [60]. A recent study also showed that the transcription factor *CDF4* induces the accumulation of ABA, which downregulates the transcript of *CAT2*, leading to the inhibition of H_2_O_2_ scavenging [61]. We posit that the levels of *FMO1*, ABA, and CAT2 interlinked by feedback loops can adapt to drought stress.

**Figure 8:**
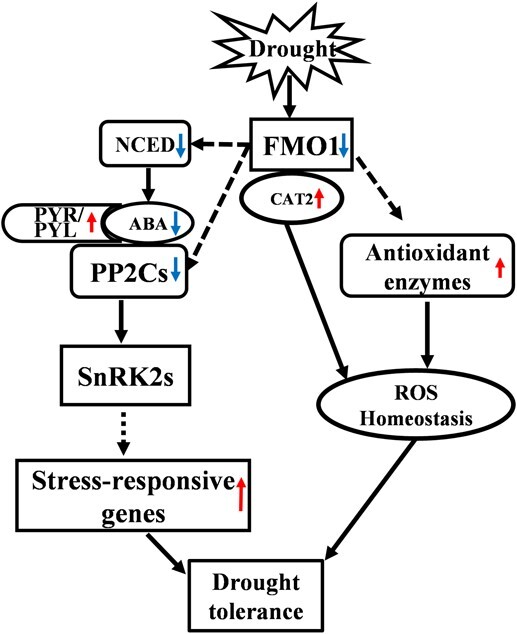
Proposed model of *FMO1*-mediated tomato plant responses to drought stress**.** When plants are subjected to drought stress, the *FMO1* is rapidly downregulated. The *FMO1* regulate the expression of *PP2C*s, *NCED*, and antioxidant enzyme genes. The FMO1 directly interacted with CAT2 promote the ROS scavenging, conferring drought tolerance by maintaining ROS homeostasis. Downregulation of *FMO1* trigger the ABA-dependent signaling pathway, leading to induction of stress-responsive genes, which in turn increases the drought tolerance of plants. Blue arrows indicate down-regulation, and red arrows mean up-regulation or/and increased activity or content.

We elucidated the mechanisms through which *FMO1* negatively regulated the drought tolerance of tomato. When the plants coped with drought, *FMO1* silencing inhibited the expression of *PP2Cs* to induce the response of the ABA signaling pathway. Meanwhile, the ABA biosynthesis gene *NCED1* downregulated and inhibited ABA biosynthesis. Thus, the ABA content decreased. *FMO1* directly interacted with CAT2 to promote ROS scavenging, thereby conferring drought tolerance by improving the activities of CAT and POD to eliminate ROS and reduce membrane lipid peroxidation. Therefore, *FMO1* downregulation triggered the ABA-dependent signaling pathway, leading to the induction of stress-responsive genes and drought tolerance (Fig. 8). In summary, *FMO1* knockdown in tomato provided enhanced drought tolerance by regulating the ABA signaling and CAT2-mediated pathways maintaining ROS homeostasis. Thus, *FMO1* is a novel target gene in genetic engineering for the enhancement of plant drought tolerance.

## Material and methods

### Bioinformatics analysis and transgenic plant generation

The coding sequence of *FMO1* was PCR-amplified from tomato (*Solanum lycopersicum*) cDNA. The tomato *FMO1* and its homologous protein sequences were compared using Clustal W2. *FMO1*-OE and *FMO1*-RNAi vectors were constructed as previously described [31]. In brief, *FMO1* was cloned into pMV to yield *FMO1*-OE (under the CaMV35S promoter). The pHellsgate 2 vector (Invitrogen, CA, USA) was used for *FMO1*-RNAi construction via a recombination reaction using a 390 bp fragment. After genetic transformation to tomato M82, the resulting positive *FMO1*-OE and *FMO1*-RNAi plants were analysed through qPCR. The transgenic plants with significantly increased (*FMO1*-OE) or decreased (*FMO1*-RNAi) transcripts were selected for further analysis. The *SlCAT2* OE and RNAi plants were obtained from a previously study [49]. The primers are listed in Table S6 (see online supplementary material).

### Plant treatments and quantitative real-time PCR

The tissue and plant growth regulator treatments were established as described previously [62]. In brief, WT seedlings at the five-leaf stage were used for the treatments. The leaves were sprayed with ABA (100 μM), GA (100 μM), ethephon (100 μM; an Eth releaser), JA (100 μM), SA (100 μM), and MV (100 μM) for the plant growth regulator treatment. The soil was irrigated with PEG (100 mM) and NaCl (100 mM) for the drought and salt treatment, and the control was sprayed with distilled water.

RNA extraction and cDNA synthesis were performed as described previously [62]. qRT-PCR reactions were implemented with the SYBR Green Real-time PCR system (Bao Guang, China), and the PCR products were monitored using the CFX96 PCR system.

### Abiotic stress assays

The *FMO1* transgenic plants with significant differential expression were selected for drought stress assays. We used a mannitol-simulated drought and applied NaCl for high-salinity treatments on MS medium. The seeds were germinated in MS medium, and the uniformly sprouted seedlings were sub-cultured in MS media containing 200 mM mannitol and NaCl for the treatments. No-stress treatment was used as the control, with at least four replicates per treatment. Drought tolerance testing was performed on the soil-grown transgenic plants as previously described [30], and a water loss assay was performed as previously described [31]. The MDA and Pro contents and CAT and POD activities were measured as described in a previous study [63]. Histochemical staining by DAB and NBT after drought stress was conducted as described previously [63].

### Transcriptome analysis

The leaves of WT, *FMO1* OE, and Ri lines under 17-day drought and normal watering were used for RNA extraction. After reverse transcriptase, we found that cDNA libraries of WT, OE, and Ri were present in DT and CK (labeled WTCK, OECK, RiCK, WTDT, OEDT, and RiDT; two biological replicates per treatment). The resulting cDNA libraries were sequenced using the Illumina NovaSeq platform (Nova, China). After sequencing, the filtered reads were aligned to the tomato genome (ITAG3.0). The mapped reads, differential expression, and gene enrichment were analysed as described previously [64].

### Yeast screening library and analysis

The *SlFMO1* gene sequence was amplified from tomato cDNA and ligated to pGBKT7 by enzyme digestion to obtain the pGBKT_7_-*FMO1* (pBD-*FMO1*) fusion vector. The pBD-*FMO1* was used as bait to screen the tomato cDNA library by following the manufacturer’s instructions (Clontech, USA). In brief, pBD-*FMO1* and pGADT_7_ recombinant tomato homogeneous cDNA library plasmids were co-transformed into yeast then transferred to SD/−Trp-Leu-His-Ade media for screening. Fifty randomly positive clones were randomly selected, sequenced, and analysed.

### Determination of ABA content

The ABA levels were determined by Nanjing Convinced-test Technology Co., Ltd. The leaves of the WT, *FMO1*-OE, and *FMO1*-Ri plants were sampled at 17 days from the DT and CK plants and ground to powder form in liquid nitrogen. The samples (1 g each) were transferred to 15 mL screw-cap tubes. Then, 8 μL (1 μg/mL) of ABA internal standards (Sigma, USA) were added to each 15 mL tube. ABA extraction and HPLC–ESI–MS/MS analysis (Q-EXACTIVE LC–MS System, USA) were performed as previously described [65].

### Co-IP and BiFC assays

The coding sequences of *SlFMO1* and *SlCAT2* were fused into the pEarleyGate202-YC vector and pEarleyGate201-YN vector (http://www.biovector.net/), respectively. The resulting pEarleyGate202-SlFMO1-YC-Flag and pEarleyGate201-CAT2-YN-HA constructs were transformed into *Agrobacterium* GV3101. Then, *Agrobacterium* samples containing pEarleyGate202-SlFMO1-YC-Flag and pEarleyGate201-CAT2-YN-HA constructs were mixed (1:1) or separately infiltrated into *N. benthamiana* leaves. Protein extraction reagents (50 mM Tris–HCl, pH 7.5, 1% polyvinylpolypyrrolidone, 150 mM NaCl, 5 mM EDTA, 10% glycerol, 2 mM dithiothreitol, 1 mM PMSF, and 1× protease inhibitor cocktail) were used for protein extraction. After centrifugation at 12000 g at 4°C for 15 min, 30–50 μl of the supernatant was obtained as input, and an equal volume of 2× SDS loading dye (SDS-PAGE) was added for control. For Co-IP assays of SlFMO1 and CAT2, Flag-tagged SlFMO1 was co-infiltrated with SlCAT2-HA. The protein supernatants were extracted with 1 mL of wash buffer (50 mM Tris–HCl, pH 7.5, 10% glycerol, 150 mM NaCl, 1 mM PMSF, and 5 mM EDTA) incubated with 10 μL of anti-HA-tag magnetic beads. All input and IP samples were separated via 10% SDS-PAGE then detected by immunoblotting using anti-HA (1:1000) or anti-Flag (1:1000) antibodies. Goat anti-mouse IgG (1:100000) was employed as a secondary antibody.

The BiFC assays was conducted as previously described [31]. In brief, *N. benthamiana* leaves were infiltrated with GV3101 harboring the resulting FMO1: YFP^N^ and SlCAT2: YFP^C^ constructs that were mixed at a ratio of 1:1. After 48 h of infiltration, the samples were observed under a confocal laser microscope (Zeiss, LSM780, Germany).

## Acknowledgements

We thank Dr Long Cui (Huazhong Agriculture University) for affording the overexpression and RNAi knockdown tomato seeds of *SlCAT2,* professor Khurram Ziaf (University of Agriculture Faisalabad-Pakistan) for revising this manuscript, and Sharing Platform of Large-Scale Instruments & Equipments in Academy of Agricultural Science, Southwest University. This work was supported by grants from the National Natural Science Foundation of China (No. 31872123), and Natural Science Foundation of Chongqing, China (No. cstc2019jcyj-msxmX0333) the Fundamental Research Funds for the Central Universities (No. XDJK2020B060).

## Author contributions

L.W. and Y.Z. carried out most of the experiments; L.W. and Y.D. helped to analyse the data. C.C., X.C. and N.S. helped to test the drought resistance; J.L., X.Z, and Y.P. designed the research; J.L. and Y.Z. wrote the paper.

## Data availability

The RNA-Seq raw data is available in Sequence Read Archive (accession number PRJNA869132). Other relevant data are presented within the paper and its supplementary files.

## Conflict of interest

None declared.

## Supplementary data

Supplementary data is available at *Horticulture Research* online.

## Supplementary Material

Web_Material_uhad037
