# Supplementary material for: Novel flavin-containing monooxygenase protein FMO1 interacts with CAT2 to negatively regulate drought tolerance through ROS homeostasis and ABA signaling pathway in tomato
Source: Hortic Res. 2023 Feb 28;10(4):uhad037. doi: 10.1093/hr/uhad037 (PMC10124749; doi:10.1093/hr/uhad037)
Supplement: Web_Material_uhad037 [file web_material_uhad037.zip › Supplemental Table S1 Summary of RNA-Seq sample sequencing data quality..docx]

Supplemental Table S1 Summary of RNA-Seq sample sequencing data quality.

| sample | raw_reads | clean_reads | clean_bases | error_rate | Q20 | Q30 | GC_pct |
| --- | --- | --- | --- | --- | --- | --- | --- |
| \| CKRi1 \| \| --- \| \| CKRi2 \| \| CKOE1 \| \| CKOE2 \| \| CKWT1 \| \| CKWT2 \| \| DTRi1 \| \| DTRi2 \| \| DTOE1 \| \| DTOE2 \| \| DTWT1 \| \| DTWT2 \| | \| 45751042 \| \| --- \| \| 43447500 \| \| 46163034 \| \| 42678412 \| \| 47045642 \| \| 44060104 \| \| 44025448 \| \| 42482974 \| \| 43767830 \| \| 41262710 \| \| 41005552 \| \| 41963356 \| | \| 43978682 \| \| --- \| \| 42026094 \| \| 44431884 \| \| 40966390 \| \| 46114430 \| \| 41171074 \| \| 42543614 \| \| 40821586 \| \| 42155888 \| \| 39932444 \| \| 39676194 \| \| 39799190 \| | \| 6.6G \| \| --- \| \| 6.3G \| \| 6.66G \| \| 6.14G \| \| 6.92G \| \| 6.18G \| \| 6.38G \| \| 6.12G \| \| 6.32G \| \| 5.99G \| \| 5.95G \| \| 5.97G \| | \| 0.02 \| \| --- \| \| 0.02 \| \| 0.02 \| \| 0.02 \| \| 0.02 \| \| 0.02 \| \| 0.02 \| \| 0.02 \| \| 0.02 \| \| 0.02 \| \| 0.02 \| \| 0.02 \| | \| 98.15 \| \| --- \| \| 98.19 \| \| 98.11 \| \| 98.18 \| \| 98.82 \| \| 98.18 \| \| 98.24 \| \| 98.22 \| \| 98.14 \| \| 98.11 \| \| 98.19 \| \| 98.16 \| | \| 94.41 \| \| --- \| \| 94.6 \| \| 94.38 \| \| 94.53 \| \| 95.98 \| \| 94.55 \| \| 94.65 \| \| 94.6 \| \| 94.48 \| \| 94.42 \| \| 94.59 \| \| 94.49 \| | \| 42.38 \| \| --- \| \| 42.44 \| \| 42.42 \| \| 42.39 \| \| 42.3 \| \| 42.39 \| \| 42.51 \| \| 42.58 \| \| 42.2 \| \| 42.33 \| \| 42.41 \| \| 42.29 \| |
