# Supplementary material for: Novel flavin-containing monooxygenase protein FMO1 interacts with CAT2 to negatively regulate drought tolerance through ROS homeostasis and ABA signaling pathway in tomato
Source: Hortic Res. 2023 Feb 28;10(4):uhad037. doi: 10.1093/hr/uhad037 (PMC10124749; doi:10.1093/hr/uhad037)
Supplement: Web_Material_uhad037 [file web_material_uhad037.zip › Supplemental Table S2 Statistics of RNA-Seq sample and reference genome comparison.docx]

Supplemental Table S2 Statistics of RNA-Seq sample and reference genome comparison.

| sample | total_reads | total_map | unique_map | multi_map |
| --- | --- | --- | --- | --- |
| \| CKRi1 \| \| --- \| \| CKRi2 \| \| CKOE1 \| \| CKOE2 \| \| CKWT1 \| \| CKWT2 \| \| DTRi1 \| \| DTRi2 \| \| DTOE1 \| \| DTOE2 \| \| DTWT1 \| \| DTWT2 \| | \| 43978682 \| \| --- \| \| 42026094 \| \| 44431884 \| \| 40966390 \| \| 46114430 \| \| 41171074 \| \| 42543614 \| \| 40821586 \| \| 42155888 \| \| 39932444 \| \| 39676194 \| \| 39799190 \| | \| 42637295(96.95%) \| \| --- \| \| 40751991(96.97%) \| \| 43199143(97.23%) \| \| 39837461(97.24%) \| \| 44959731(97.5%) \| \| 39835544(96.76%) \| \| 41411334(97.34%) \| \| 39697617(97.25%) \| \| 40755196(96.68%) \| \| 38665619(96.83%) \| \| 38573757(97.22%) \| \| 38688997(97.21%) \| | \| 42022532(95.55%) \| \| --- \| \| 40158253(95.56%) \| \| 42568414(95.81%) \| \| 39229397(95.76%) \| \| 44305745(96.08%) \| \| 39280013(95.41%) \| \| 40814890(95.94%) \| \| 39113962(95.82%) \| \| 40141599(95.22%) \| \| 38060339(95.31%) \| \| 38028005(95.85%) \| \| 38142581(95.84%) \| | \| 614763(1.4%) \| \| --- \| \| 593738(1.41%) \| \| 630729(1.42%) \| \| 608064(1.48%) \| \| 653986(1.42%) \| \| 555531(1.35%) \| \| 596444(1.4%) \| \| 583655(1.43%) \| \| 613597(1.46%) \| \| 605280(1.52%) \| \| 545752(1.38%) \| \| 546416(1.37%) \| |
