# Supplementary material for: Novel flavin-containing monooxygenase protein FMO1 interacts with CAT2 to negatively regulate drought tolerance through ROS homeostasis and ABA signaling pathway in tomato
Source: Hortic Res. 2023 Feb 28;10(4):uhad037. doi: 10.1093/hr/uhad037 (PMC10124749; doi:10.1093/hr/uhad037)
Supplement: Web_Material_uhad037 [file web_material_uhad037.zip › Supplemental Table S3 Selected DEGs from RNA-Seq for real-time PCR verification.docx]

Supplemental Table S3 Selected DEGs from RNA-Seq for real-time PCR verification.

|  |  | Fragments Per Kilobase of exon model per Million mapped fragments（FPKM） | | | | | |  |
| --- | --- | --- | --- | --- | --- | --- | --- | --- |
| number | Gene name | CKRi | CKWT | CKOE | DTRi | DTWT | DTOE | Gene function description or family |
| 1 | Solyc03g096670.3 | 18.35 | 19.09 | 20.63 | 130.62 | 404.88 | 505.45 | PP2C1 |
| 2 | Solyc07g062970.3 | 25.95 | 25.92 | 35.59 | 48.58 | 117.49 | 178.84 | PP2C2 |
| 3 | Solyc05g052980.3 | 50.47 | 67.19 | 63.96 | 312.76 | 570.02 | 577.52 | PP2C2 |
| 4 | Solyc07g040990.3 | 33.70 | 32.76 | 43.56 | 101.32 | 228.84 | 238.33 | PP2C4 |
| 5 | Solyc03g121880.3 | 92.20 | 95.17 | 111.44 | 268.28 | 387.84 | 354.76 | PP2C5 |
| 6 | Solyc01g100040.3 | 94.76 | 88.94 | 100.50 | 151.53 | 210.46 | 194.22 | PP2C6 |
| 7 | Solyc12g005200.2 | 5.70 | 5.64 | 7.93 | 7.27 | 14.37 | 22.63 | Electron transfer flavoprotein |
| 8 | Solyc07g056570.1 | 16.39 | 30.51 | 7.36 | 87.33 | 312.06 | 457.41 | NCED1 |
| 9 | Solyc02g082760.3 | 241.54 | 189.56 | 222.80 | 133.85 | 43.18 | 46.67 | catalase isozyme 2 isoform X1 |
| 10 | Solyc06g005160.3 | 757.28 | 731.59 | 871.09 | 309.99 | 127.61 | 226.43 | L-ascorbate peroxidase 1 |
| 11 | Solyc11g018550.3 | 22.07 | 24.07 | 20.62 | 13.09 | 9.73 | 7.47 | thylakoid-bound ascorbate peroxidase 6 |
| 12 | Solyc02g087190.1 | 25.77 | 22.87 | 17.32 | 10.30 | 6.76 | 8.06 | PREDICTED: peroxidase 63-like |
