# Supplementary material for: Novel flavin-containing monooxygenase protein FMO1 interacts with CAT2 to negatively regulate drought tolerance through ROS homeostasis and ABA signaling pathway in tomato
Source: Hortic Res. 2023 Feb 28;10(4):uhad037. doi: 10.1093/hr/uhad037 (PMC10124749; doi:10.1093/hr/uhad037)
Supplement: Web_Material_uhad037 [file web_material_uhad037.zip › Supplemental Table S4 Partly representative DEGs related to abiotic stress from RNA-Seq.docx]

Supplemental Table S4 Partly representative DEGs related to abiotic stress from RNA-Seq.

|  | FPKM | | | | | |  |
| --- | --- | --- | --- | --- | --- | --- | --- |
| Gene name | CKRi | CKWT | CKOE | DTRi | DTWT | DTOE | Gene description |
| Solyc06g005160.3 | 757.28 | 731.59 | 871.09 | 309.99 | 127.61 | 226.43 | Cytosolic ascorbate peroxidase 1 |
| Solyc09g009390.3 | 69.53 | 66.02 | 66.89 | 58.96 | 43.04 | 39.59 | Monodehydroascorbate reductase 1 |
| Solyc01g006300.3 | 297.01 | 242.60 | 422.75 | 358.39 | 291.14 | 335.68 | Peroxidase |
| Solyc02g082760.3 | 241.54 | 189.56 | 222.80 | 133.85 | 43.18 | 46.67 | Catalase isozyme 2 isoform X1 |
| Solyc01g067740.3 | 119.16 | 175.92 | 76.21 | 146.14 | 219.80 | 414.07 | Superoxide dismutase |
| Solyc01g086960.3 | 395.44 | 333.71 | 365.78 | 272.83 | 371.74 | 504.28 | Stress-associated protein 1 |
| Solyc09g075210.3 | 74.56 | 68.42 | 66.43 | 178.23 | 252.52 | 68.24 | Heat shock protein |
| Solyc11g069090.2 | 907.73 | 878.31 | 851.78 | 909.68 | 678.57 | 541.30 | ABC transporter F family member 1 |
| Solyc04g055120.3 | 52.35 | 47.10 | 54.54 | 44.92 | 51.74 | 52.21 | Peroxisomal ABC transporter 1 |
| Solyc02g084850.3 | 6.40 | 7.92 | 6.30 | 841.63 | 5499.59 | 10151.98 | ABA stress-inducible protein TAS14 |
| Solyc12g013620.2 | 64.51 | 61.75 | 78.23 | 196.03 | 512.35 | 817.91 | Jasmonic acid 2 |
| Solyc04g076380.3 | 159.77 | 140.32 | 156.17 | 151.04 | 171.82 | 179.32 | NADPH--cytochrome P450 reductase |
| Solyc11g066440.2 | 64.18 | 62.72 | 96.69 | 76.31 | 111.74 | 125.48 | Pheophorbide a oxygenase |
| Solyc03g119060.3 | 70.26 | 88.07 | 53.69 | 87.76 | 114.31 | 81.33 | Alpha-dioxygenase 2 enzyme |
| Solyc05g052520.3 | 24.58 | 19.27 | 16.34 | 8.72 | 9.24 | 14.35 | PP2C-type phosphatase AP2C3 |
