# Supplementary material for: Novel flavin-containing monooxygenase protein FMO1 interacts with CAT2 to negatively regulate drought tolerance through ROS homeostasis and ABA signaling pathway in tomato
Source: Hortic Res. 2023 Feb 28;10(4):uhad037. doi: 10.1093/hr/uhad037 (PMC10124749; doi:10.1093/hr/uhad037)
Supplement: Web_Material_uhad037 [file web_material_uhad037.zip › Supplemental Table S5 Screening of proteins interacting with FMO1 by yeast two-hybrid.docx]

Supplemental Table S5 Screening of proteins interacting with FMO1 by yeast two-hybrid

| Gene ID | Occurance (clones) | functional annotation |
| --- | --- | --- |
| Solyc02g085950.2 | 10 | Ribulose bisphosphate carboxylase small chain |
| Solyc03g034220.2 | 8 | Ribulose bisphosphate carboxylase small chain |
| Solyc01g009420.2 | 5 | Bifunctional polymyxin resistance arnA protein |
| Solyc01g108600.2 | 5 | Presequence protease, mitochondrial |
| Solyc12g094620.1 | 4 | Catalase cat2 |
| Solyc03g078490.2 | 2 | UDP-glucuronosyltransferase |
| Solyc02g069100.2 | 2 | Cathepsin B |
| Solyc01g010750.2 | 1 | Stress responsive protein |
