# Supplementary material for: Novel flavin-containing monooxygenase protein FMO1 interacts with CAT2 to negatively regulate drought tolerance through ROS homeostasis and ABA signaling pathway in tomato
Source: Hortic Res. 2023 Feb 28;10(4):uhad037. doi: 10.1093/hr/uhad037 (PMC10124749; doi:10.1093/hr/uhad037)
Supplement: Web_Material_uhad037 [file web_material_uhad037.zip › Supplemental Table S6. List of primers used in this study.docx]

Supplemental Table S6. List of primers used in this study

| Primer name | Type | Usage |
| --- | --- | --- |
| OE FMO-F | GTGACCATGGCTTTAATCTCTTC | Construction of *FMO* overexpression vector |
| OE FMO-R | TGTCTATTGCTATTCGAGGGAAA | Construction of *FMO* overexpression vector |
| RNAi FMO-F | GGGGACAAGTTTGTACAAAAAAGCAGGCT  GTATCCGCTGAGGTGGTGAT | Construction of *FMO -*RNAi vector |
| RNAi FMO-R | GGGGACCACTTTGTACAAGAAAGCTGGGT  GCGACTGGCCAATCTCTAAC | Construction of *FMO -*RNAi vector |
| yeFMO-F | GAATTCGTGACCATGGCTTTAATCTCTTC | Construction of *FMO* yeast vector |
| yeFMO-R | GGATCCTGTCTATTGCTATTCGAGGGAAA | Construction of *FMO* yeast vector |
| NPTⅡ-F | AGACAATCGGCTGCTCTGAT | Positive detection of transgenic plants |
| NPTⅡ-R | TCATTTCGAACCCCAGAGTC | Positive detection of transgenic plants |
| ACtin-F | GTCCTCTTCCAGCCATCCAT | Internal reference primers |
| ACtin-R | ACCACTGAGCACAATGTTACCG | Internal reference primers |
| qFMO-F | GGACAGCCATATGAACCAAAAG | QRT-PCR primers |
| qFMO-R | CCTTGGTTTTAGAAGCAGGAAC | QRT-PCR primers |
| T_7_-F | TAATACGACTCACTATAGGGC | Primer for yeast plaque detection |
| AD-R | AGATGGTGCACGATGCACAG | Primer for yeast plaque detection |
| CDS III | AAATTCTAGAGGCCGAGGCG | Primer for yeast plaque detection |
| SMART III | AAGCAGTGGTATCAACGCAGAG | Primer for yeast plaque detection |
| pEarleyGate201-YN-CAT2-F | ACAAGTTTGTACAAAAAAATGATTGTGACACAGTATCGC | Co-IP vector construction |
| pEarleyGate201-YN-CAT2-R | CACCACTTTGTACAAGAACATTGTAGGCTTCACAGTGAGA | Co-IP vector construction |
| pEarleyGate202-YC-FMO1-F | ACAAGTTTGTACAAAAAAATGGCTTTAATCTCTTCAATTTTACAACATGC | Co-IP vector construction |
| pEarleyGate202-YC-FMO1-R | CACCACTTTGTACAAGAAATGTCCGTCTCTTTTAGCTTG | Co-IP vector construction |
| q1-F | CACAGTTCGGATAATGTTAGCG | QRT-PCR verification |
| q1-R | ATGTCCAGAACACCAACTTAGT | QRT-PCR verification |
| q2-F | AATTTTGCATCATGACTGGAGG | QRT-PCR verification |
| q2-R | CTTACCTCTTCCGGTATCTGAC | QRT-PCR verification |
| q3-F | CCGTGTTATATATTGGGACGGA | QRT-PCR verification |
| q3-R | CAAACACTCATCTTCGTTGGTT | QRT-PCR verification |
| q4-F | GAAATGATGTTTGTACCCCGAG | QRT-PCR verification |
| q4-R | TTTTGTGCCAGAGAAGAATTCG | QRT-PCR verification |
| q5-F | GAGATGAACAAATCACCGCTAC | QRT-PCR verification |
| q5-R | GTCAAAGAAGGTTCGAGAATCG | QRT-PCR verification |
| q6-F | AGTTGCACATGCCTTTAAGAAG | QRT-PCR verification |
| q6-R | TGATTTGGTGGATGGAAGTGTA | QRT-PCR verification |
| q7-F | CCTTGGATGAAGATGAGTCAGT | QRT-PCR verification |
| q7-R | ACCAGTTACTACAATACGTGCA | QRT-PCR verification |
| q8-F | CCCGATTTGGTATTCTGGATAAGTA | QRT-PCR verification |
| q8-R | GAGACGGATTTCGGATAAAACACT | QRT-PCR verification |
| q9-F | GCTGTCAAGTTTTACACCAGAG | QRT-PCR verification |
| q9-R | GAACTTCATTCCATCACGGATG | QRT-PCR verification |
| q10-F | CTAACCCTTCATCTCATTTGCG | QRT-PCR verification |
| q10-R | CTGGTCCATCTTTCGTGTATCT | QRT-PCR verification |
| q11-F | TACTGGGACAAAAGGTGAGATC | QRT-PCR verification |
| q11-R | TCACTGTTCCCTCGCATATAAA | QRT-PCR verification |
| q12-F | CAGATCACTTCACCTACTACCG | QRT-PCR verification |
| q12-R | GATAGGTTGATTTCTGCGTCAC | QRT-PCR verification |
