# Supplementary material for: Novel flavin-containing monooxygenase protein FMO1 interacts with CAT2 to negatively regulate drought tolerance through ROS homeostasis and ABA signaling pathway in tomato
Source: Hortic Res. 2023 Feb 28;10(4):uhad037. doi: 10.1093/hr/uhad037 (PMC10124749; doi:10.1093/hr/uhad037)
Supplement: Web_Material_uhad037 [file web_material_uhad037.zip › Supplementary information summary.docx]

**Supplementary information**

**Supplemental Figure S1** Phenotypic differences between *FMO1* transgenic lines and WT tomatoes on MS medium (7 days after sowing) and/under simulated drought and NaCl treatment (12 days after sowing).

**Supplemental Figure S2** GO analysis of DEGs in Ri, WT, and OE under DT. BP: biological process, CC: cellular component, MF: molecular function.

**Supplemental Figure S3** Scatter diagram of enrichment of the differentially expressed gene annotated to the KEGG.

**Supplemental Figure S4** Heat map of PYR/PRLs (a), WRKY (b) and LEAs (c) in GO analysis. It showing their transcriptional abundance in *FMO1* transgenic lines (OE and Ri) at DT and CK. The colored bar indicates FPKM values.

**Supplemental Figure S5** qRT-PCR analysis of the expression of selected DEGs from RNA-seq (detail gene information list in Supplemental Table S3).

**Supplemental Figure S6** Multiple sequence alignment of SlCAT2, AtCAT2 and AtCAT1.

**Supplemental Figure S7** The full blot images of Co-IP assays showing the interaction of FMO1 and CAT2 in *N. benthamiana* leaves. Anti-HA and anti-FLAG antibodies were used for immunoprecipitation respectively.

**Supplemental Table S1** Summary of RNA-Seq sample sequencing data quality.

**Supplemental Table S2** Statistics of RNA-Seq sample and reference genome comparison.

**Supplemental Table S3** Selected DEGs from RNA-Seq for real-time PCR verification.

**Supplemental Table S4** Partly representative DEGs related to abiotic stress from RNA-Seq.

**Supplemental Table S5** Screening of proteins interacting with FMO1 by yeast two-hybrid.

**Supplemental Table S6** List of primers used in this study.
