# Supplementary figures and images for: Novel flavin-containing monooxygenase protein FMO1 interacts with CAT2 to negatively regulate drought tolerance through ROS homeostasis and ABA signaling pathway in tomato
Source: Hortic Res. 2023 Feb 28;10(4):uhad037. doi: 10.1093/hr/uhad037 (PMC10124749; doi:10.1093/hr/uhad037)

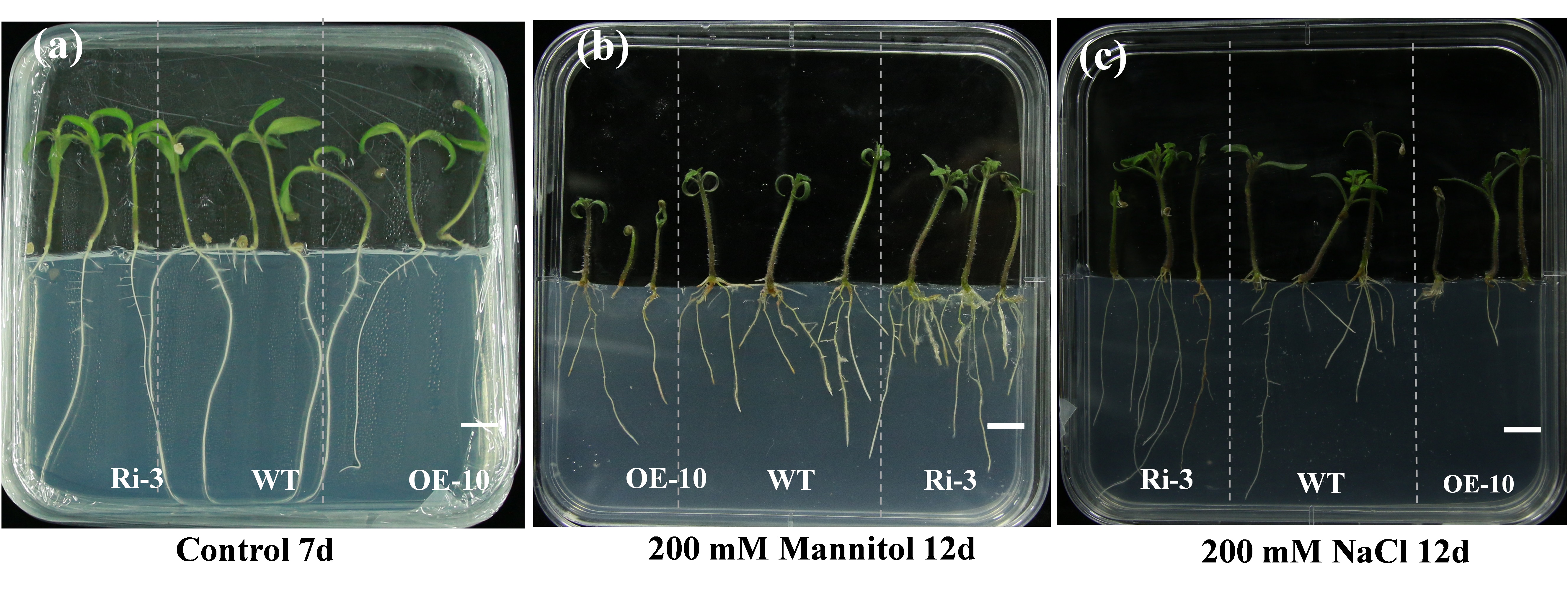

Supplement: Web_Material_uhad037 [file web_material_uhad037.zip › Supplemental Figure S1 Phenotypic differences between FMO1 transgenic lines and WT tomatoes on MS medium under simulated drought and NaCl treatment .jpg]

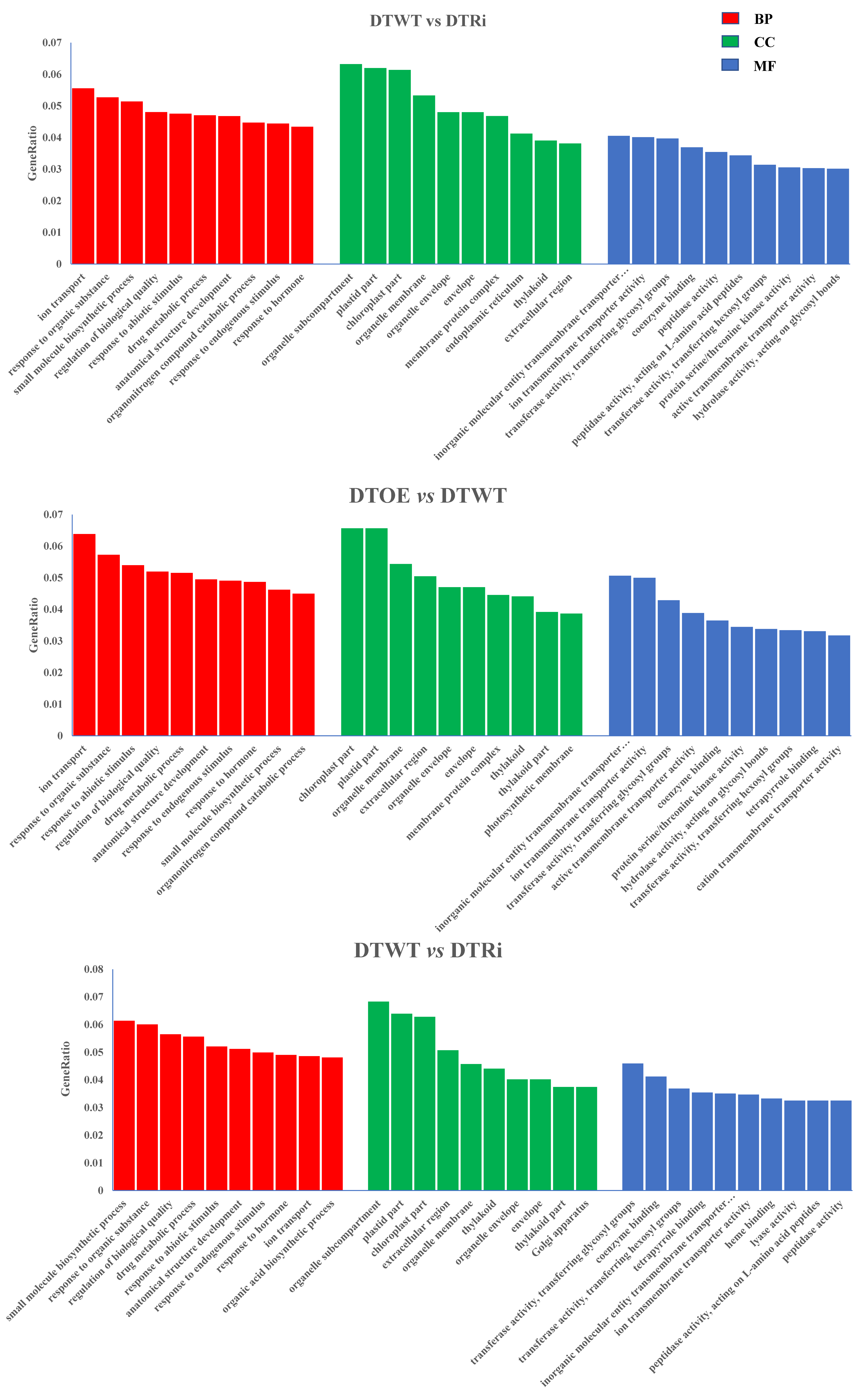

Supplement: Web_Material_uhad037 [file web_material_uhad037.zip › Supplemental Figure S2 GO analysis of DEGs in Ri, WT, and OE under DT (2).tif]

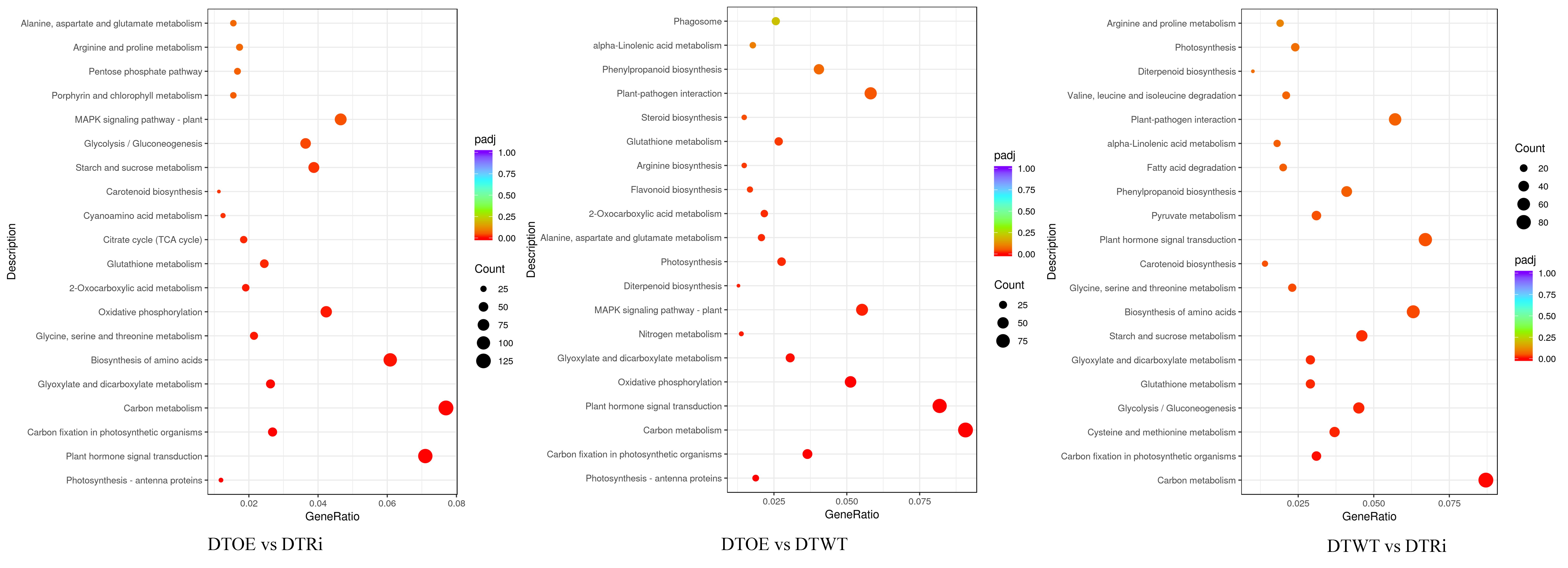

Supplement: Web_Material_uhad037 [file web_material_uhad037.zip › Supplemental Figure S3 Scatter diagram of enrichment of the differentially expressed gene annotated to the KEGG.tif]

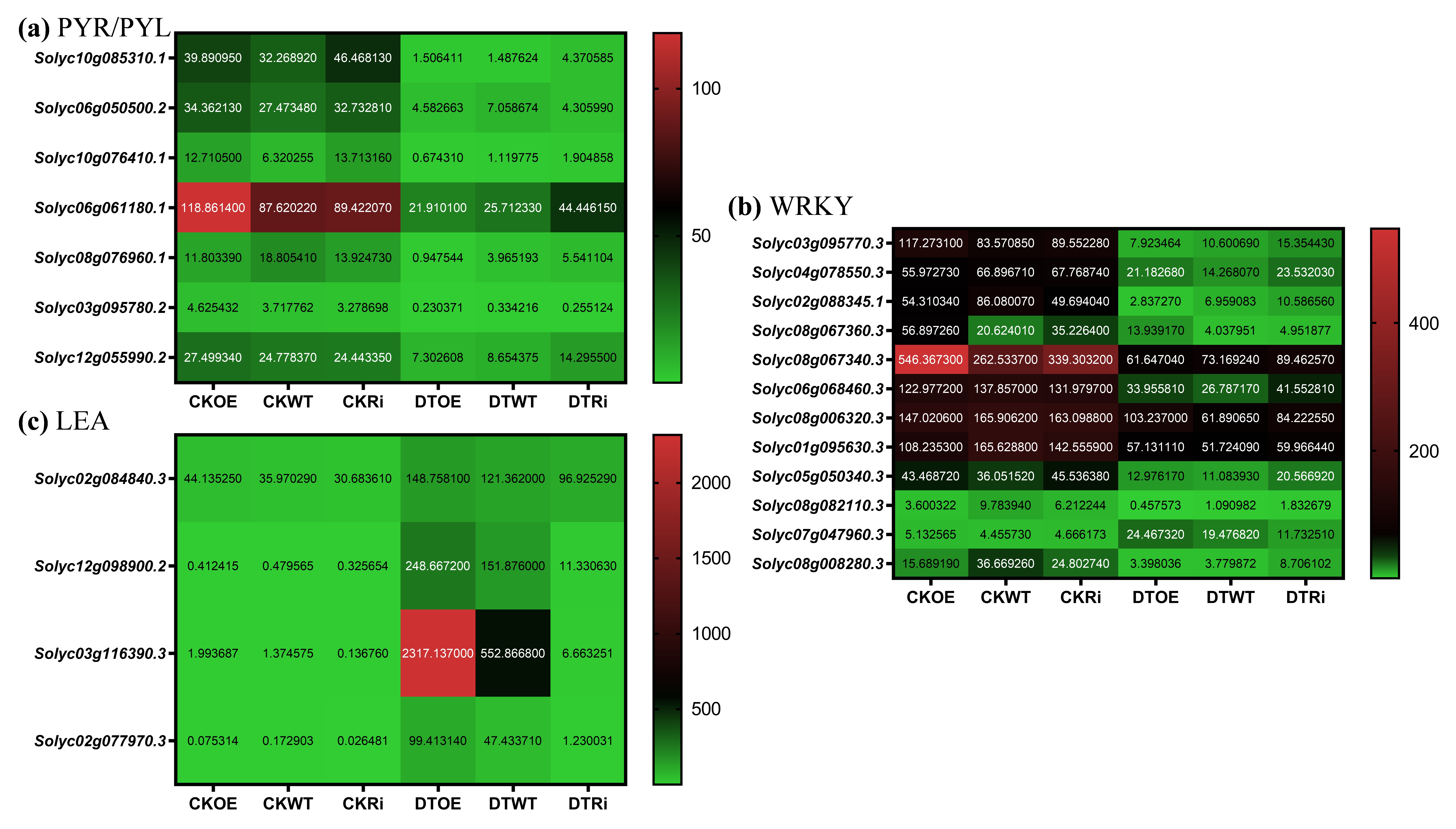

Supplement: Web_Material_uhad037 [file web_material_uhad037.zip › Supplemental Figure S4 Heat map of PYRPRLs (a), WRKY (b) and LEAs (c) in GO analysis.jpg]

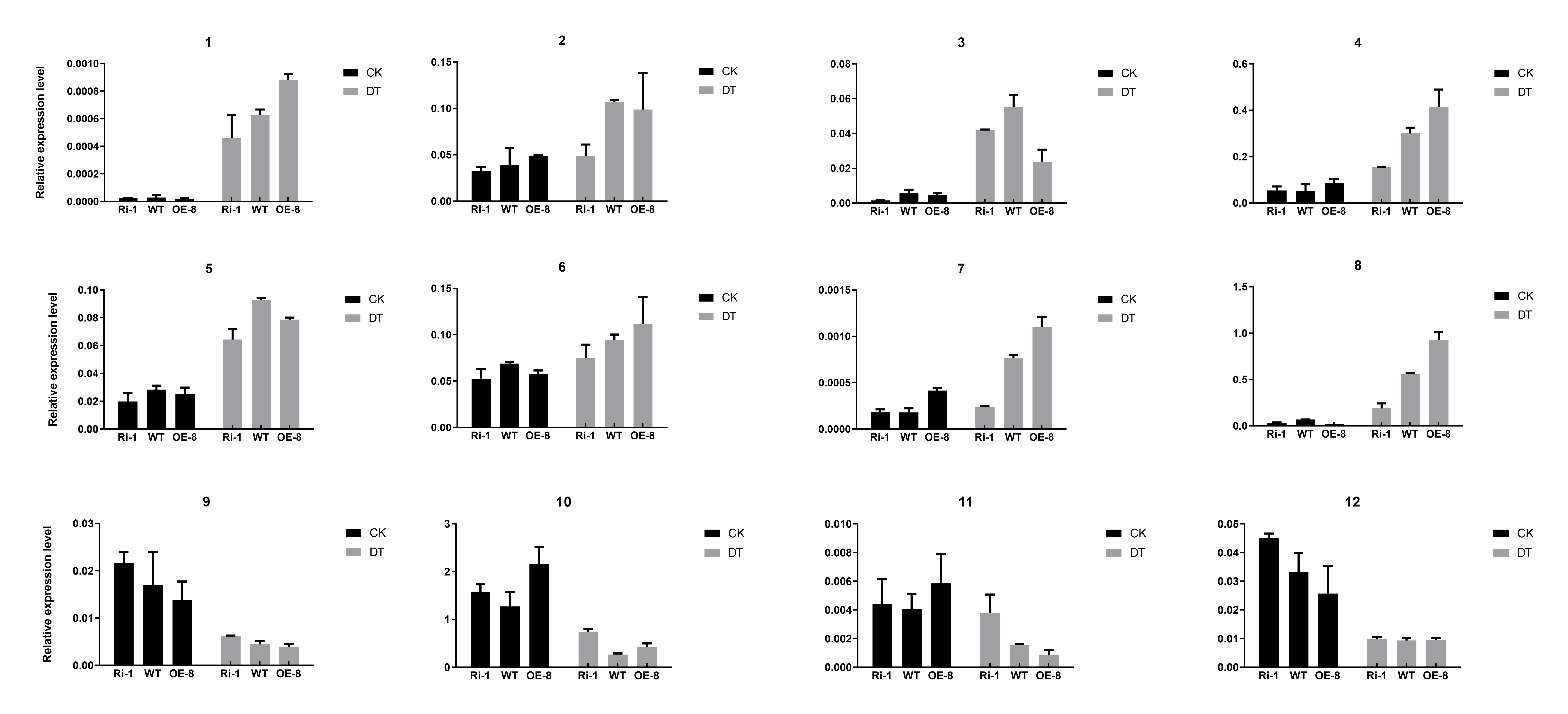

Supplement: Web_Material_uhad037 [file web_material_uhad037.zip › Supplemental Figure S5 qRT-PCR analysis of the expression of selected DEGs from RNA-seq.jpg]

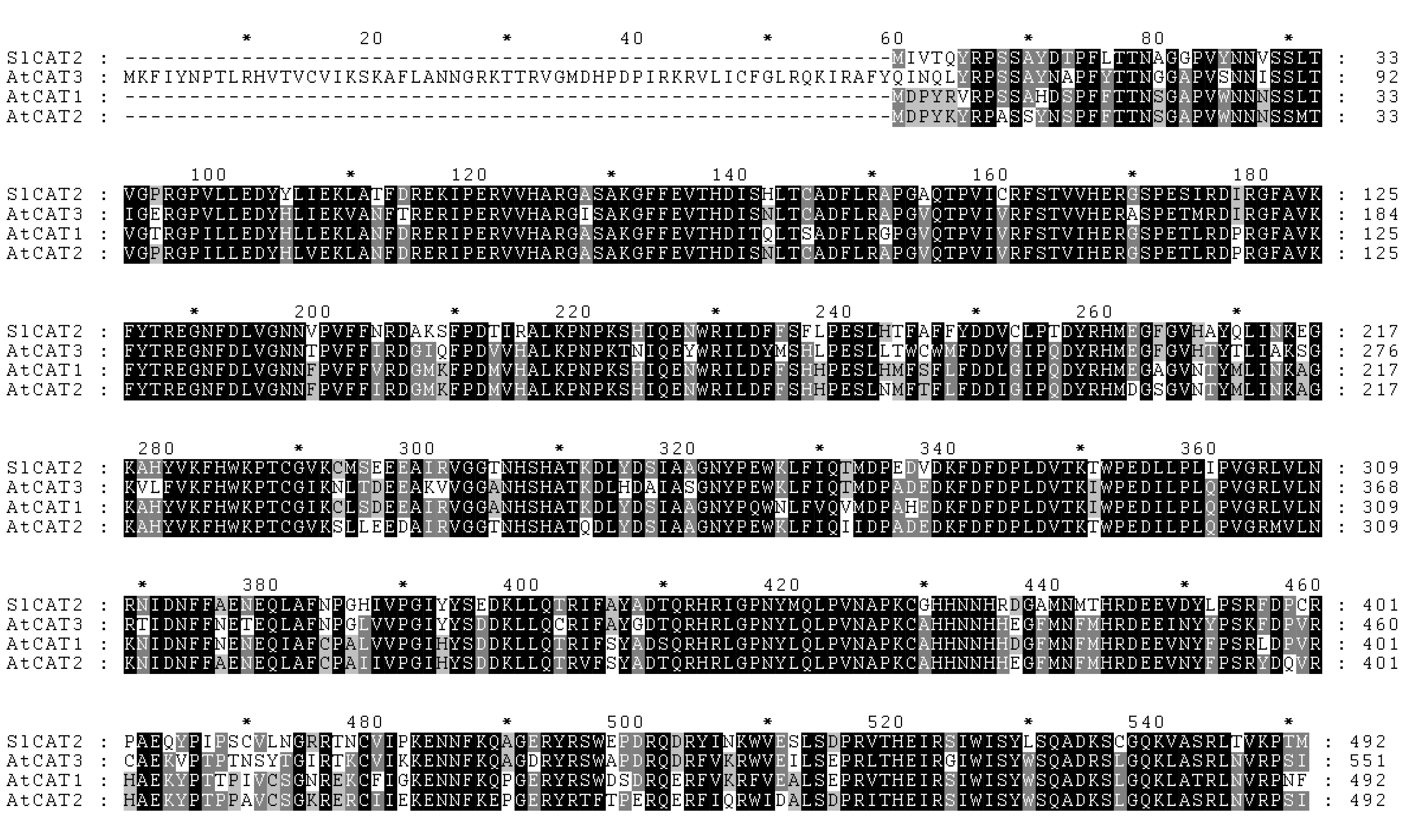

Supplement: Web_Material_uhad037 [file web_material_uhad037.zip › Supplemental Figure S6 Multiple sequence alignment of SlCAT2, AtCAT2 and AtCAT1.jpg]

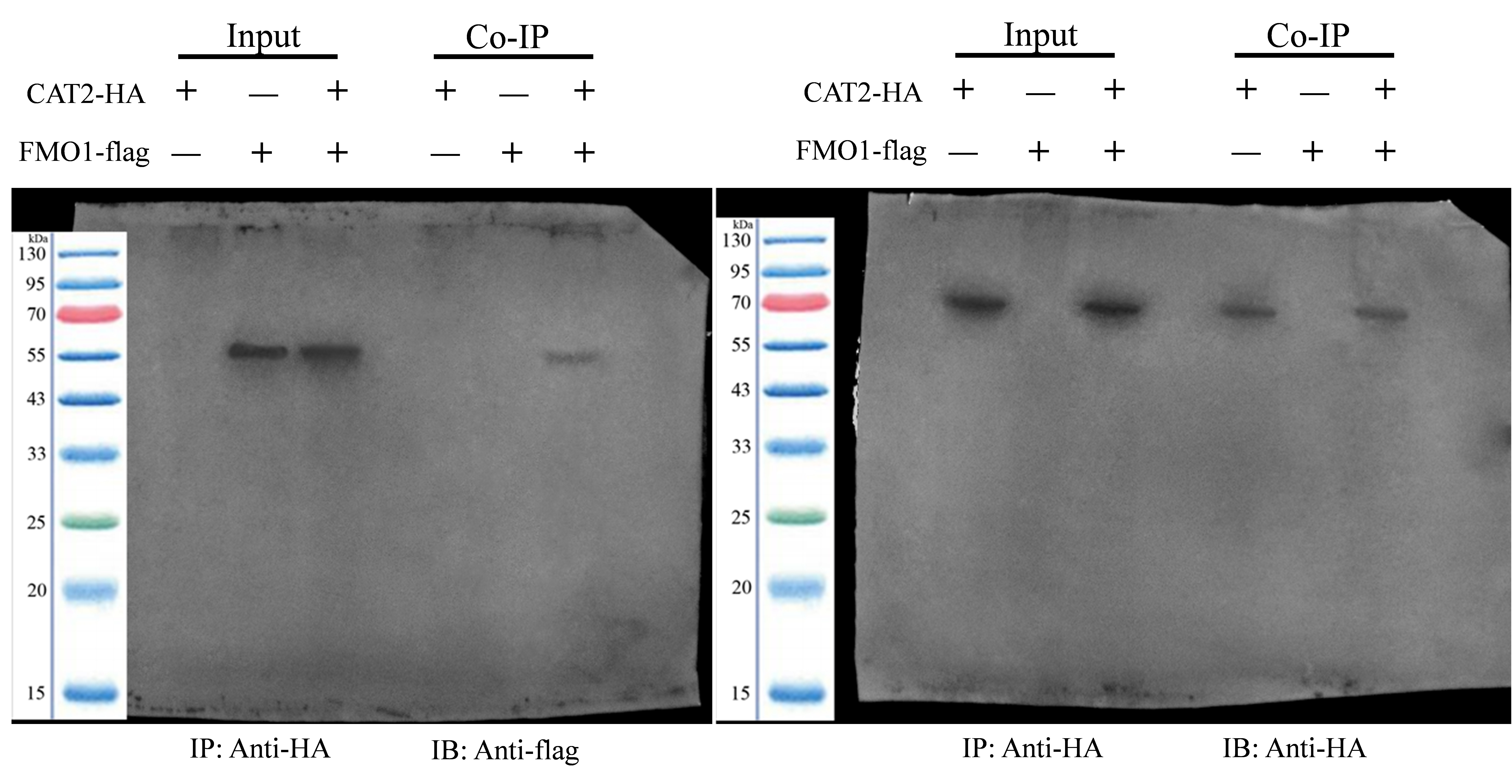

Supplement: Web_Material_uhad037 [file web_material_uhad037.zip › Supplemental Figure S7 The full blot images of Co-IP assays showing the interaction of FMO1 and CAT2 in N. benthamiana leaves.jpg]
